# Isotopic and proteomic evidence for communal stability at Pre-Pottery Neolithic Jericho in the Southern Levant

**DOI:** 10.1038/s41598-023-43549-1

**Published:** 2023-09-29

**Authors:** Xiaoran Wang, Baoshuai Zhang, Yufeng Sun, Tara Ingman, Stefanie Eisenmann, Mary Lucas, Erin Scott, Jana Ilgner, Gao Wu, Petrus le Roux, Xiaotong Wu, Xingxiang Zhang, Anchuan Fan, Patrick Roberts, Philipp W. Stockhammer

**Affiliations:** 1 Institute for Pre- and Protohistoric Archaeology and Archaeology of the Roman Provinces, Ludwig Maximilians University, 80539 Munich, Germany; 2 Department of Archaeogenetics, Max Planck Institute for Evolutionary Anthropology, 04103 Leipzig, Germany; 3 USTC Archaeometry Laboratory, University of Science and Technology of China, 96 Jinzhai Road, Hefei, 230026 China; 4 Department of Anthropology, Washington University in St. Louis, 1 Brookings Drive, Louis, MO 63130 USA; 5 Koç University Research Center for Anatolian Civilizations (ANAMED), Istanbul, 34433 Turkey; 6 Faculty of Theology, Humboldt University of Berlin, 10178 Berlin, Germany; 7 Department of Archaeology, Max Planck Institute of Geoanthropology, 07745 Jena, Germany; 8 Core Facility Center for Life Sciences, University of Science and Technology of China, Hefei, 230026 China; 9 Department of Geological Sciences, University of Cape Town, Rondebosch, 7701 South Africa; 10 School of History, Renmin University of China, Beijing, 100872 China; 11 isoTROPIC Research Group, Max Planck Institute of Geoanthropology, 07745 Jena, Germany

**Keywords:** Archaeology, Archaeology, Biological anthropology

## Abstract

As one of the key, long-term occupied sites in the Southern Levant, Jericho was one of the most important early Neolithic centres to witness social and economic changes associated with the domestication of plants and animals. This study applies strontium (^87^Sr/^86^Sr), oxygen (δ^18^O) and carbon (δ^13^C) isotope analyses to the enamel of 52 human teeth from Pre-Pottery Neolithic (PPN) layers of Jericho to directly study human diet and mobility and investigate the degree of consolidation and the flexibility of social organization of Jericho society in the PPN period. The results indicate only two non-local individuals out of the 44 sampled inhabitants identified by strontium isotope analysis and are consistent with the presence of a largely sedentary community at PPN Jericho with no evidence for large-scale migration. We also construct strontium spatial baselines (^87^Sr/^86^Sr map) with local ^87^Sr/^86^Sr signatures for the sites across the Southern Levant based on systematic compilation and analysis of available data. In addition, we apply proteomic analysis of sex-specific amelogenin peptides in tooth enamel for sex estimation of the sampled individuals (n = 44), the results of which showed a sex-biased ratio (more male than female detected in this sample pool) in Jericho society during the PPN period, which may be due to the limited sample size or selective ritual practices like particular burial zones used for specific groups. We also pretreated a batch of human bone samples recovered from PPNB Jericho for stable carbon and nitrogen isotope analyses for dietary investigations. However, the extracted collagen showed poor preservation and no valid δ^13^C or δ^15^N data were obtained.

## Introduction

Neolithization, defined here as the process of a semi- or complete transition to agriculture with an expected concordant gradual move to sedentism^1–5^, took place asynchronously in different communities in different regions of the Near East^6–8^. In contrast to initial ideas of a “Neolithic Revolution” emanating from a single region^9^, multiple centers of experimentation with plant cultivation and animal herding have been identified across the Levant, southeastern Anatolia and the Zagros Mountains in the early Holocene^10,11^, with the pace, pattern and mechanisms of domestication and spread of farming, as well as associated cultural changes, being highly variable^2,4,12–14^. It has been demonstrated that, from the Pre-Pottery Neolithic A (PPNA) onwards, people in the Southern Levant tended to live more sedentary lifestyles and that this change potentially started much earlier here in comparison to other regions of the Fertile Crescent (FC). This has been supported by recent strontium isotopic research in PPN Southeastern Anatolia at Nevali Çori which shows that the inhabitants did not become sedentary until the Middle Pre-Pottery Neolithic B (PPNB)^15^. To better understand the pace of sedentism in different regions of the FC and its relationship to economic and social changes associated with the domestication of plants and animals, it is essential to undertake multidisciplinary approaches to palaeodiet and palaeomobility in different parts of the FC.

**Figure 1:**
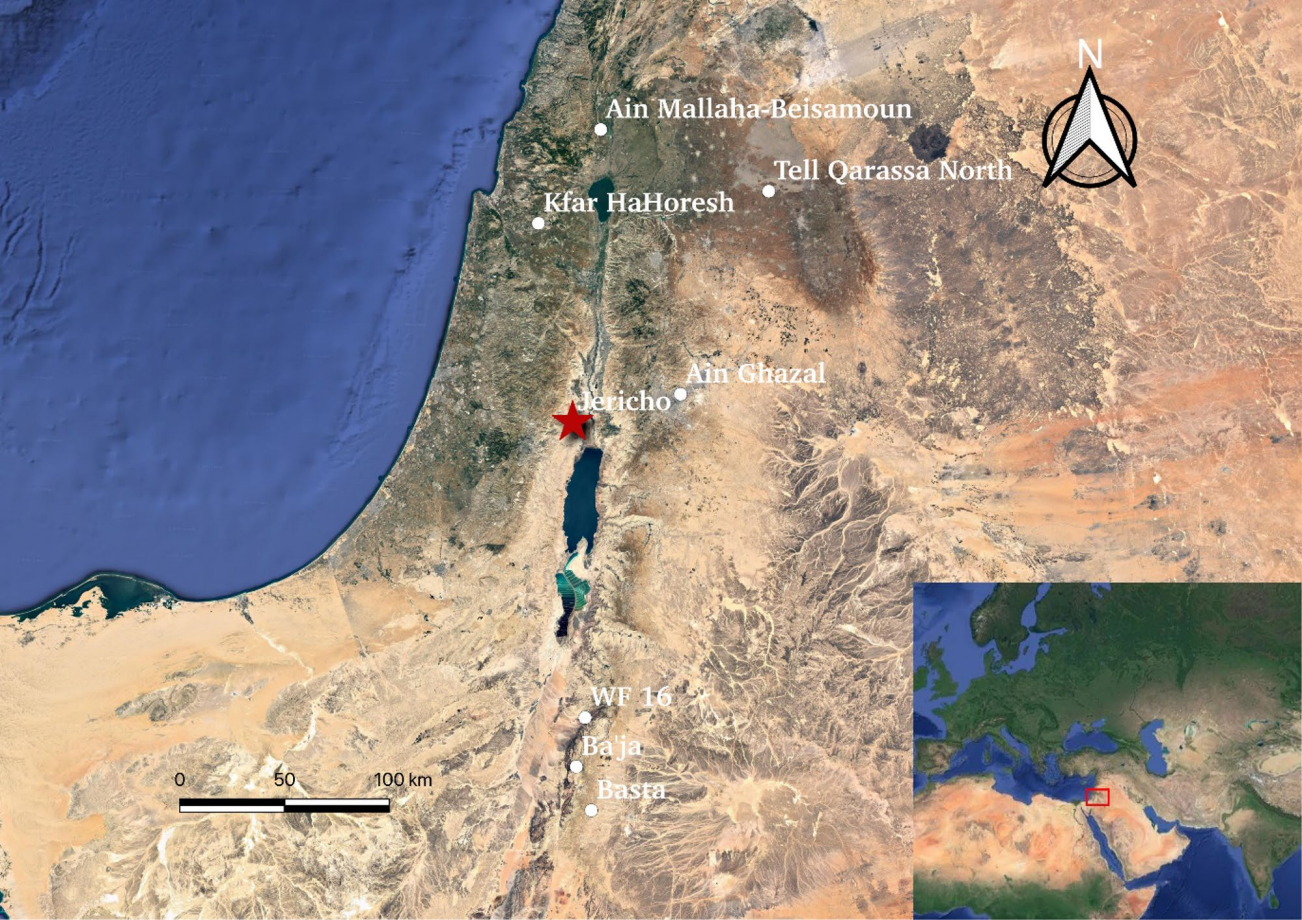
The location of Jericho and the relevant sites mentioned in the study (map illustration was carried out with QGIS 3.28 LTR, the URL link: https://qgis.org/downloads/macos/qgis-macos-ltr.dmg).

Located at today’s Tell es-Sultan, to the north of the Dead Sea in the Jordan Valley at N31°52′15″ E35°26′35″ (Fig. 1), ancient Jericho records over 10,000 years of human history, from the Late Natufian (10,500–8500 BC) to the Ottoman period (1516–1918 AD), without any apparent significant breaks in occupation^16–19^. The terminology of PPNA and PPNB, the transition between which is marked by changes in lithic industry, domestic architecture, and economic strategies, was first defined by Kenyon during her excavations at Jericho^20^. Since early excavations in the mid-twentieth century, Jericho has become one of the most emblematic sites of the PPN in the Southern Levant and has been a key site for understanding the Southern Levant during the PPNA (ca. 8500–7500 BC) and PPNB (ca. 7500–6000 BC) in particular^11,21,22^. Thus, Jericho provides a focal case study for testing the nature and timing of the social transition from highly mobile to more sedentary communities, as well as its relationship, or not, to plant cultivation and animal herding. So far, there is no clear evidence for animal domesticates during the PPNA, while the domestication of sheep and goats is evident in the succeeding PPNB levels^23–25^. From a frequently-used camp site for Natufian groups^20,26^, the large scale of the site and the appearance of a stone tower that is over 8.5 m high and the walls dating to roughly 8300 BC in the PPNA make Jericho distinct from other contemporary sites in the Southern Levant in this period. However, despite decades of archaeological excavation, direct insights into ancient mobility and diet, as well as social structure, based on the biological materials recovered from Jericho is still lacking.

The function of the tower and the walls remains debated—they may have potentially served as defensive fortifications^27^ or flood prevention barriers^28^, as well as having possible communal or ritual significance according to some more recent research^29^. These large communal structures are potentially testimony to a society more sedentary than those that came before. Jericho has also yielded rare insights into PPN burial practices, where individuals were mostly interred under floors or in rubble fills, and collective burials were common^17,18,30^. A noteworthy mortuary custom was a ritual practice in the form of skull removal and skull plastering, with or without the mandibles included. Several skull caches have been uncovered at the site and are assumed to have served a kind of ancestral veneration or memorial function^31,32^. Although Jericho, to date, has preserved the highest numbers of plastered skulls in the region, this treatment of crania or statues of the dead is not unique to the site but is rather a common regional phenomenon during the PPN. Furthermore, unplastered skulls are found even more widely across nearly the entire Southern Levant^32^. This shared ritual system across the PPN Levantine sphere, and the imported materials/products seem to indicate interaction and connections between Jericho and other communities, perhaps driven by human mobility. For example, the discovery of prestige items, such as obsidian from Anatolian sources, indicates a wide exchange network with spheres beyond the immediate environs that were already active since the Natufian period, continuing into the PPNB^33–35^. However, isotopic research of human remains from PPN periods that can provide direct evidence of human mobility and migration is still limited, in contrast with more focus on earlier materials dating to Natufian phases, e.g.,^36,37^.

To shed more light on ancient mobility at PPN Jericho, we sampled human remains for multiple bio-archaeological analyses. ^87^Sr/^86^Sr ratios of tooth enamel derive from the geological context of an area and its contribution to an individual’s water and food intake during enamel formation^38,39^. We sampled 52 human enamel samples from PPN Jericho for ^87^Sr/^86^Sr analysis, as well as one archaeological animal sample (JCH079.A, see Table 1 and Dataset S1) and modern plant samples collected from near Jericho (the coordinates are listed in the Dataset S4) to evaluate the local bioavailable ^87^Sr/^86^Sr range. To use strontium as a geochemical tracer for archaeological biomaterials, a baseline of bioavailable ^87^Sr/^86^Sr in the region must be determined^40^ (SI Appendix Note S2.1). A growing number of isotope studies on materials recovered from the ancient Levant have been reported in previous publications. Therefore, we also compiled the available ^87^Sr/^86^Sr signatures published in relevant research to construct strontium spatial baselines (^87^Sr/^86^Sr map) across the Southern Levant, providing access to comparable data for future studies of archaeological, paleoenvironmental and paleoclimatic research in this area^41–45^. We also measured δ^18^O values from the human tooth enamel samples as an independent record of an individual’s water intake which, through their relationship to local temperatures, altitude, continentality and other environmental effects^46,47^, allow them to also act as a geographical tracers. In addition, we applied δ^13^C analysis to tooth enamel, to gain direct insights into diet (SI Appendix Note S2.2.1). Finally, because sex determination of the sampled individuals in the absence of both the complete skeletons for osteological identification and aDNA extraction for shotgun-genomic sex estimation was challenging, proteomics analysis was used, to determine biological sex for the sampled individuals from PPN Jericho^48,49^.

**Table 1:** The ^87^Sr/^86^Sr ratios of the samples from Jericho measured in this study.

Sample ID	Material	Date	87Sr/86Sr	± 2SD internal
JCH003.C	Human dental enamel	PPNB	0.708004	0.000014
JCH004.C	Human dental enamel	PPNB	0.707998	0.000010
JCH011.C	Human dental enamel	PPNB	0.708069	0.000012
JCH015.B	Human dental enamel	PPNB	0.708011	0.000013
JCH019.B	Human dental enamel	PPNA	0.708014	0.000012
JCH042.B	Human dental enamel	PPNB	0.708145	0.000012
JCH042.C	Human dental enamel	PPNB	0.708064	0.000011
JCH053.B	Human dental enamel	PPNB	0.708058	0.000011
JCH054.C	Human dental enamel	PPNA	0.708163	0.000011
JCH055.B	Human dental enamel	PPNB	0.708037	0.000011
JCH056.B	Human dental enamel	PPNB	0.708065	0.000010
JCH057.B	Human dental enamel	PPNB	0.708062	0.000011
JCH058.B	Human dental enamel	PPNB	0.708089	0.000011
JCH058.C	Human dental enamel	PPNB	0.708050	0.000012
JCH059.B	Human dental enamel	PPNB	0.708060	0.000012
JCH060.A	Human dental enamel	PPNB	0.708011	0.000016
JCH060.B	Human dental enamel	PPNB	0.708018	0.000010
JCH060.C	Human dental enamel	PPNB	0.708020	0.000008
JCH061.B	Human dental enamel	PPNB	0.709341	0.000012
JCH061.D	Human dental enamel	PPNB	0.708497	0.000011
JCH062.A	Human dental enamel	PPNB	0.707972	0.000012
JCH062.C	Human dental enamel	PPNB	0.708006	0.000013
JCH063.A	Human dental enamel	PPNB	0.708043	0.000011
JCH063.E	Human dental enamel	PPNB	0.708077	0.000012
JCH064.A	Human dental enamel	PPNB	0.708015	0.000010
JCH064.B	Human dental enamel	PPNB	0.708017	0.000011
JCH065.B	Human dental enamel	PPNA	0.708025	0.000013
JCH066.C	Human dental enamel	PPNB	0.708003	0.000011
JCH067.A	Human dental enamel	PPNB	0.707954	0.000011
JCH068.B	Human dental enamel	PPNB	0.708064	0.000010
JCH069.A	Human dental enamel	PPNB	0.708034	0.000009
JCH070.B	Human dental enamel	PPNB	0.707980	0.000011
JCH071.B	Human dental enamel	PPNB	0.708027	0.000013
JCH072.B	Human dental enamel	PPNB	0.707974	0.000010
JCH074.A	Human dental enamel	PPNB	0.708102	0.000010
JCH075.A	Human dental enamel	PPNB	0.708146	0.000012
JCH076.B	Human dental enamel	PPNB	0.708160	0.000012
**JCH079.A**	**Fauna dental enamel**	**PPNB**	**0.707988**	**0.000010**
JCH084.B	Human dental enamel	PPNB	0.708407	0.000012
JCH086.B	Human dental enamel	PPNB	0.708061	0.000012
JCH087.C	Human dental enamel	PPNB	0.707999	0.000012
JCH089.A	Human dental enamel	PPNB	0.708104	0.000013
JCH092.A	Human dental enamel	PPNB	0.708087	0.000009
JCH093.C	Human dental enamel	PPNB	0.708073	0.000011
JCH096.A	Human dental enamel	PPNB	0.707905	0.000009
JCH097.A	Human dental enamel	PPNB	0.708153	0.000012
JCH099.A	Human dental enamel	PPNB	0.708132	0.000015
JCH100.B	Human dental enamel	PPNB	0.707974	0.000012
JCH103.B	Human dental enamel	PPNB	0.707978	0.000011
JCH104.C	Human dental enamel	PPNB	0.708007	0.000012
JCH105.B	Human dental enamel	PPNB	0.708126	0.000012
JCH106.B	Human dental enamel	PPNB	0.708114	0.000010
JCH107.B	Human dental enamel	PPNB	0.708062	0.000013
**ZY-11156**	**Saltworts**	**Modern**	**0.708069**	**0.000012**
**ZY-11157**	**Saltworts**	**Modern**	**0.708040**	**0.000010**
**ZY-11158**	**Saltworts**	**Modern**	**0.708073**	**0.000012**
**ZY-11159**	**Saltworts**	**Modern**	**0.707983**	**0.000012**

The bolded samples are used to build the local bioavailable strontium isotope baseline of Jericho, including 1 archaeological fauna enamel sample and 4 modern plants. More detailed contextual information is included in the Dataset S1.

## Materials

We sampled 52 human teeth from 44 individuals from Jericho PPN, with three coming from the PPNA and the rest from the PPNB (for more details of sample information, see SI Appendix Dataset S1), for ^87^Sr/^86^Sr, δ^13^C and δ^18^O isotope analyses. Permanent 1st, 2nd and 3rd molars were the preferred dentition for sampling, representing, respectively, the ages of 0–3 years, 3–7 years, and adolescence or early adulthood, depending on individual differences in the formation time of M3^50,51^. Multiple teeth were sampled per individual, where preservation allowed, in order to evaluate possible variation reflecting mobility during the individual’s lifetime. All samples were taken from the collection curated by the late anthropologist Olav Röhrer-Ertl and are now housed in the Anthropological Collection of the University of Göttingen, Germany (for more details on the history of the excavation and subsequent itinerary of the bone material, see SI Appendix Note S1). In addition, to determine the local ^87^Sr/^86^Sr baseline, we also sampled one archaeological animal (goat/sheep) tooth and four saltwort plants collected at different localities in the surrounding arid areas of Jericho where impacts of farming and fertilizer contamination could be ruled out. More details, including the coordinates of the plant samples, are presented in Dataset S4. We analyzed sex chromosome-linked isoforms of amelogenin by nanoflow liquid chromatography-tandem mass spectrometry (nanoLC-MS/MS) to determine the sex of the sampled individuals (n = 44). The full principles and methods are provided in SI Appendix Note S2.3.

A batch of samples (n = 47) of human bone recovered from PPNB Jericho were also pretreated for stable carbon and nitrogen isotope analyses of bone collagen to investigate possible dietary patterns (Dataset S3). Collagen was successfully extracted from all samples, however, the C/N ratios of all samples indicated that the extracted collagen did not qualify for further measurement because of poor preservation, which is a common situation in the similar studies in the Near East (also referring to Richards et al.^52^), which could be the result of the use of animal glues when the bones were re-articulated during past restauration efforts. Due to the same issue, ^14^C dating was not possible on this batch of samples. Since the sampled material derived from the early excavations there was frequently a lack of correlation between the handwritten records and the archived samples. Due to the special mortuary practices performed *at situ*, some individuals were only represented by skulls^31,32^ and the contextual information of the excavated individuals was limited in the original notes, thus these individuals were not always identifiable from the archive. Overall, as absolute dates of these samples are lacking, we have to rely on the records of the original excavations and relative chronological phasing, and association with ages determined from subsequent dating of these phases elsewhere in the site. Available archaeological contexts of the sampled individuals in this study are presented in Dataset S1. We also attempted aDNA analysis on the same batch of samples (targeting the petrous bones and dentine), but preservation levels again were insufficient, and the analysis was therefore not possible. The relevant pretreatment processes and methods and the data from the relevant measurements are included in the SI Appendix Note S2.4 and Dataset S3.

## Results

### Isotope analyses

**Figure 2:**
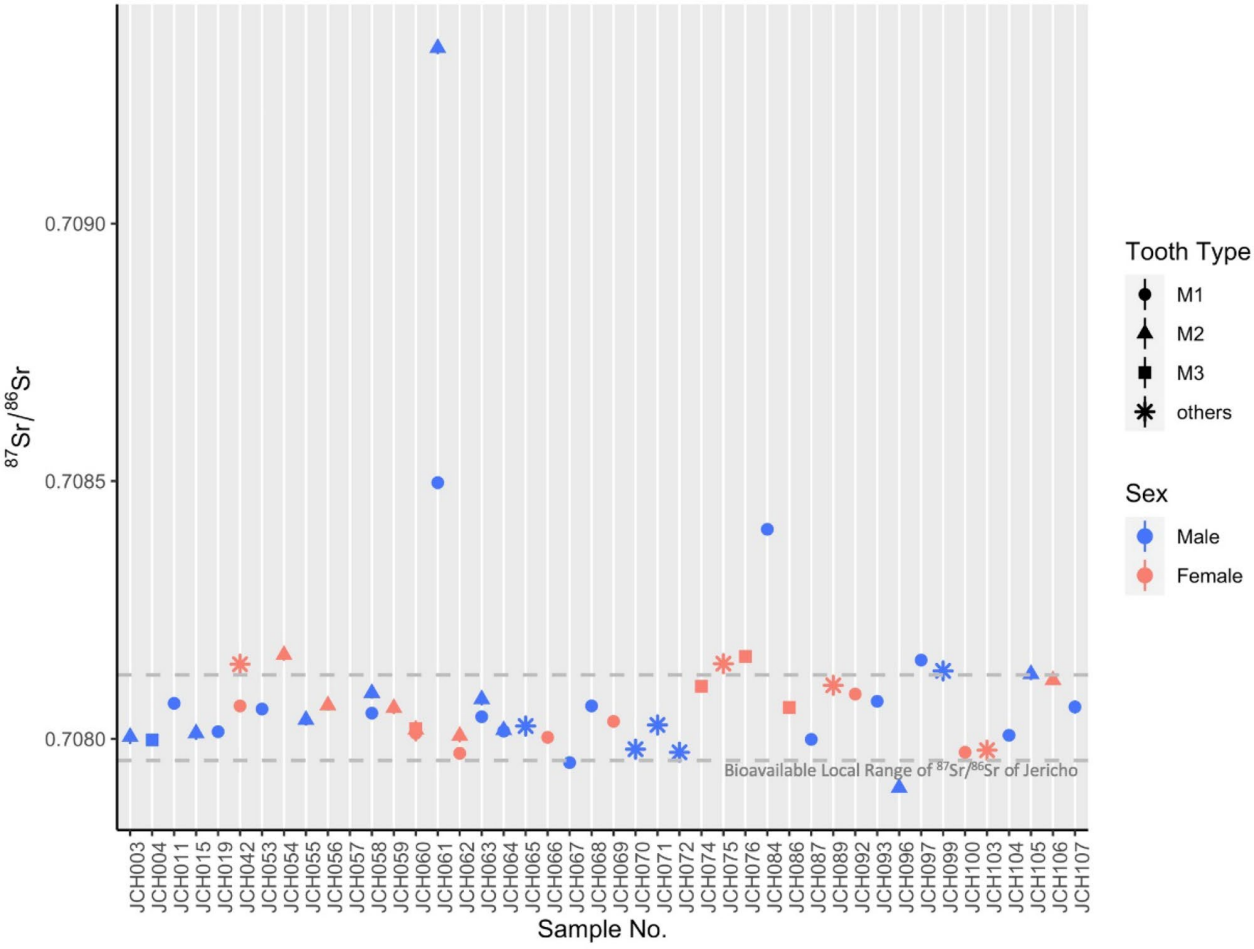
^87^Sr/^86^Sr plot of the sampled human teeth excavated from PPNA and PPNB Jericho, with multiple samples from the same individual plotted on the same axis (Dataset S1).

**Figure 3:**
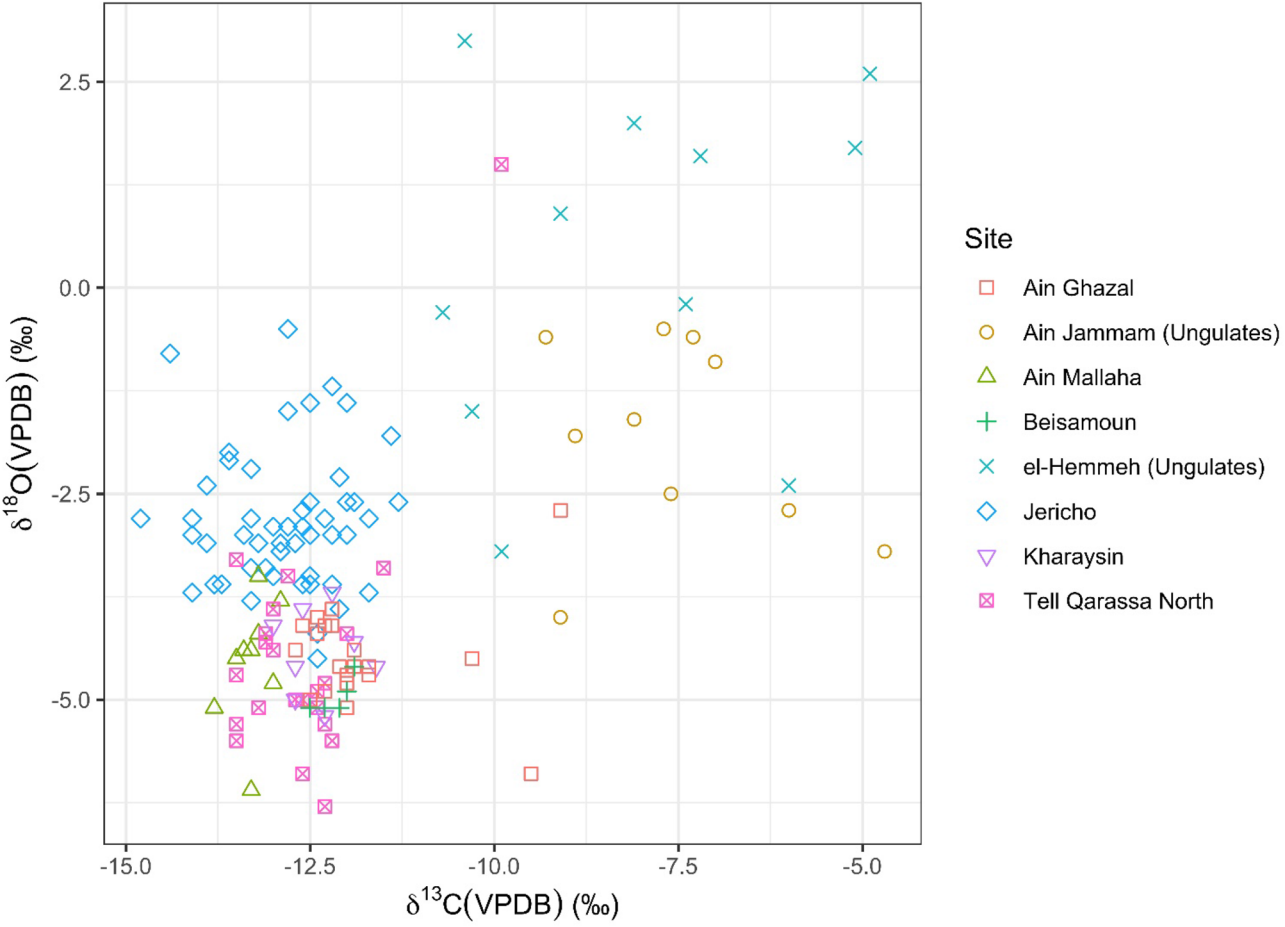
Scatter plot with δ^13^C_VPDB_ and δ^18^O_VPDB_ values (n = 51) of the individuals (n = 44) sampled from Jericho, grouping with published data from some other sites in the prehistoric (either of Natufian or PPN periods) Southern Levant (the details and references of the materials and the data included are presented in Dataset S6).

The results of the multi-isotopic analyses of all samples from Jericho are presented in Dataset S1. To summarize, we report 52 ^87^Sr/^86^Sr ratios from 44 human individuals, and five ^87^Sr/^86^Sr ratios from the environmental samples (four modern plant samples and one dental enamel of archaeological animal) (see Fig. 2, Table 1, Datasets S1, S4), as well as 51 paired δ^13^C and δ^18^O values from 43 human individuals which were also measured for ^87^Sr/^86^Sr. Regarding the δ^13^C and δ^18^O data, the δ^18^O values of all the 51 samples range from − 4.5 to − 0.5‰ with a mean of –2.8 ± 0.1‰ (2SD). Meanwhile, δ^13^C values range from − 14.8 to − 11.3‰ with an average of − 12.8 ± 0.3‰ (2SD) (Datasets S1, S6, Fig. 3).

The results of ^87^Sr/^86^Sr analysis show that most human^87^Sr/^86^Sr ratios fall within or close to local ^87^Sr/^86^Sr bioavailable values of Jericho based on the modern plant and archaeological animal samples, ranging from 0.707944 to 0.708117 (mean ± 2SD, mean = 0.708031) (more information of the environmental samples is included in Datasets S1, S4)^53^. The ^87^Sr/^86^Sr values of 42 individuals range from 0.707905 to 0.708163 with the exception of three teeth, coming from two male individuals (according to the sex determination results, see below and Table 2), JCH061 and JCH084, which had distinctively higher ^87^Sr/^86^Sr values and appear to show non-local residence at least during their childhood. Notably, JCH061 displays distinct values for both M1 (0.708497) and M2 (0.709341), which developed during his infancy and childhood respectively. Both values are much higher than the local range of Jericho, where he was buried, demonstrating potential evidence of mobility during his youth.

**Table 2:** Metadata from the proteomic analysis of sex-specific amelogenin peptides on the sampled individuals (n = 44) in this study.

Sample number	Sex	Number of total peptides	Number of AMELY unique peptides	Deamidation N (%)	Deamidation Q (%)
JCH003	M	152	13	99.49	100
JCH004	M	107	7	100	100
JCH011	M	92	3	100	100
JCH015	M	38	4	100	100
JCH019	M	93	4	100	100
JCH042	F	146	0	99.78	100
JCH053	M	67	2	100	100
JCH054	F	206	0	97.51	99.81
JCH055	M	87	9	99.11	100
JCH056	F	80	0	100	100
JCH057	M	127	8	100	97.92
JCH058	M	158	12	99.85	100
JCH059	F	40	0	82.71	92.58
JCH060	F	90	0	100	100
JCH061	M	102	4	91.09	99.35
JCH062	F	6	0	100	100
JCH063	M	133	7	100	100
JCH064	M	112	6	100	100
JCH065	M	76	4	97.65	100
JCH066	F	155	0	94.91	100
JCH067	M	53	4	100	100
JCH068	M	112	3	100	96.37
JCH069	F	197	0	100	99.46
JCH070	M	67	2	99.12	100
JCH071	M	125	16	100	100
JCH072	M	144	10	100	100
JCH074	F	99	0	98.06	100
JCH075	F	147	0	99.69	100
JCH076	F	144	0	100	100
JCH084	M	130	6	100	100
JCH086	F	82	0	100	100
JCH087	M	199	13	100	98.83
JCH089	F	227	0	100	100
JCH092	F	119	0	100	100
JCH093	M	192	14	100	99.41
JCH096	M	139	8	99.83	99.33
JCH097	M	172	12	97.83	99.89
JCH099	M	69	5	96.76	100
JCH100	F	200	0	99.77	100
JCH103	F	113	0	100	100
JCH104	M	88	4	97.50	100
JCH105	M	176	8	92.16	100.00
JCH106	F	131	0	100.00	100.00
JCH107	M	177	13	100.00	99.90

### Geological context and ^87^Sr/^86^Sr baselines of the Southern Levant

The analysis of ^87^Sr/^86^Sr ratios depends largely upon the geological variability reflected in the bioavailable ^87^Sr/^86^Sr measured for the region under study^54,55^. The geological variation of the Southern Levant is distributed in north–south bands of different geological and lithological assemblages. However, it is not the case that one zone contains only a single type of bedrock or lithology; rather, all zones consist of multiple geological components of different ages^56–58^. Moffat et al. summarized and reviewed ^87^Sr/^86^Sr data based on geological materials and archaeological case studies from Israel/the Levant covering a period from 1993 to 2020^59^. Hartman and Richards studied the relative contributions of bedrock and atmospheric sources to bioavailable strontium pools in local soils in Northern Israel and the Golan regions and produced a map of bioavailable ^87^Sr/^86^Sr ratios based on modern plant and invertebrate samples^60^.

**Figure 4:**
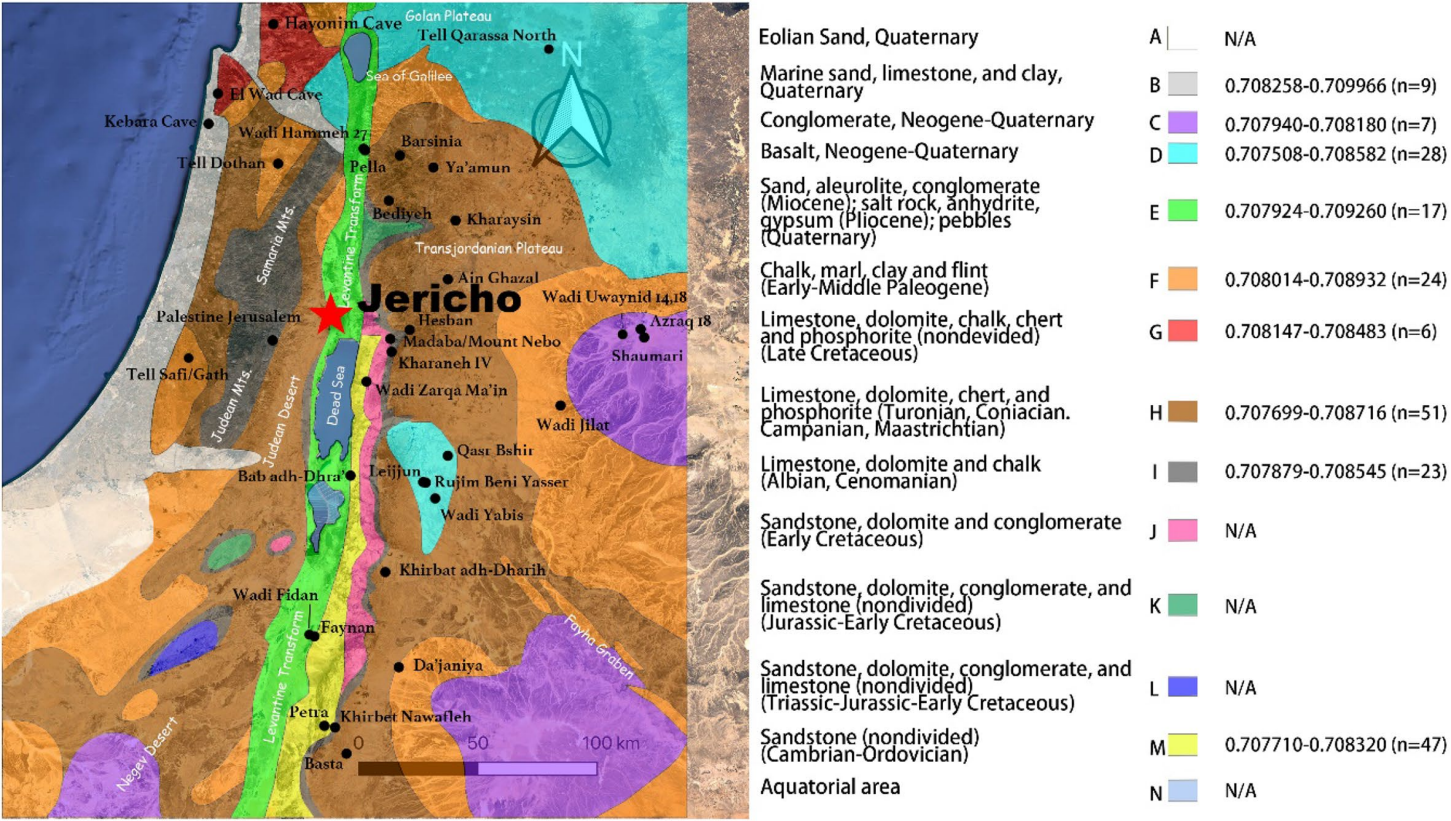
Integration of bioavailable ^87^Sr/^86^Sr intervals and geological formations across the Southern Levant, based on the published data to date and modified after^56,93^; N/A indicates that a local range could not be established due to the lack of data; detailed calculations of the included data and the corresponding references are presented in Dataset S2.

To present the spatial distribution of ^87^Sr/^86^Sr values in the Southern Levant, we compiled and analyzed the published ^87^Sr/^86^Sr ratios from 36 sites and recalculated the mean values and standard deviations of data available for each site and used the maximum range within a given geological province defined to be of generally similar geological context (presented as distinctive legends in Fig. 4)^36,37,61–72^. For the sites with more than (and including) three datapoints available we applied mean ± 2SD to calculate the local bioavailable ^87^Sr/^86^Sr range of each site. Otherwise, we use a single ratio or simply a range for the two ratios available. For bioavailable local ^87^Sr/^86^Sr, ranges of the included sites corresponding to each legend are shown in in Fig. 4 (see also Dataset S2). For more details of the principles of calculation, the details of the type and chronological context of the material used for each dataset and the coordinates of the sites see Dataset S4. The information for the other sites with published data available, but beyond the scope of this map, are presented in Dataset S5. The data included here mainly derive from materials appropriate for bioavailable strontium assessments of multiple types, including modern or archaeological fauna or plant remains, while excluding data from geological materials, soil and rock which could reflect larger variability ratios compared to biological materials^73^. We tried to avoid using bone apatite ratios due to the risk of diagenesis unless there was no other option, in which case they are marked with asterisk. The distribution and potential sources of bioavailable strontium in northern Israel and the Golan have been thoroughly studied^60^. We, therefore, do not include these in Fig. 4. Notably, our bioavailable strontium ‘map’ may not strictly follow geological distinctions, and we must consider the potential endmembers in practice that served as the bioenvironment for local inhabitants. For instance, some bioavailable local strontium signatures represent eroded or alluvial sediments much younger than the local bedrock^61^.

The geological context of the Southern Levant can be simplified to five^72^ or seven^37^ main zones: Coastal areas (Legend B), Western Highlands (Legends F, H, I), Jordan Rift Valley (Legend E), Eastern Highlands (Legends M, H), Golan Heights (Legend D), Hula Basin (Legend G), Basalt area (Legend D), Azraq Basin (Legend C) (Fig. 4). Jericho is located in the Jordan Rift Valley, the geological depression of which consists predominantly of Quaternary, sandstone, mudstone, and gravels, formed during the Miocene^56,72^. The geological context near Jericho consists of Lisan Marl diluvium which is dominated by limestone and gravel^56,74^. According to our spatial assessment of existing and measured ^87^Sr/^86^Sr data, sampling densities are unevenly available for the different zones. The most intensively investigated zone is Legend H including the Eastern Highlands and part of the Western Highlands (N (number) _sample_ = 51, N (number) _site_ = 10), followed by Legends M (N_sample_ = 47, N_site_ = 4), D (N_sample_ = 28, N_site_ = 5), I (N_sample_ = 28, N_site_ = 5) and F (N_sample_ = 24, N_site_ = 3). The southern-west and the northern-east of the Southern Levant have not been adequately investigated, and Legends A, J, K L and N are still lacking data to date. Our mapping of the data shows that the Jordan Valley fillings where Jericho is located present the widest distribution of ^87^Sr/^86^Sr ratios, spanning from 0.707924 to 0.7092260 (Legend E), with higher values in the north and lower values in the south. The other sites in the same zone of the Jordan Valley are Pella, with a lower range (0.707924–0.707967, 2SD)^61^ than Jericho, and Wadi Hammeh 27 (0.708020–0.709260, 2SD)^36^, which is generally higher, as well as Wadi Fidan with values (0.707929–0.708144, 2SD)^61^ that are close to the Jericho range (Dataset S2); there may be other possibilities not yet excavated or identified in surveys, as well.

The local bioavailable ^87^Sr/^86^Sr signature of Jericho (0.707944–0.708117, mean ± 2SD, based on the modern plant and archaeological animal samples as mentioned above) is consistent (or partly consistent) with almost all the geological zones, except for those represented by Legend B and Legend G, as well as the values documented for the region where the site sits previously. Therefore, interpretations of ^87^Sr/^86^Sr variability are challenging given the homogeneity of certain values across hundreds of kilometres as well as fine-grained variation within a matter of kilometres. This means that assessment of ‘local’ and ‘non-local’ using this approach is challenging, as is any attempt to provenance individuals. As a result, in interpreting our human data, we focus more on relative difference and inner comparison amongst the measured samples from Jericho and assessment of intra-population patterns among our datasets.

### Sex determination

Amelogenin peptides were successfully extracted from all samples. In this study, for the samples without AMELY unique peptides, the number of total peptides in all samples was larger than the reference value of 30. Therefore, we believe that the sex of the individuals identified in this study is secure. In addition, the deamidation percentage of glutamine (N) and asparagine (Q) in this study was higher than 80%, which was much higher than that of contaminants (N = 19.52%, Q = 26.49%). Moreover, no peptide related to amelogenin was found in the blank control group. Therefore, the identification of amelogenin peptides is considered reliable. Detailed results are presented in Table 2.

The sampling of individuals was randomized and the samples were from almost all the excavation units of the PPNB layers at Jericho (Dataset S1). The ratio of male to female individuals in the dataset was 27:17 (Table 2). There are several possible explanations for this bias toward male individuals. It could be a mere coincidence by which chance more males than females were selected for sampling or that the anthropological collection simply contained more males. Alternatively, the sampling accurately reflects differing burial practices for males and females leading to uneven obtaining of the samples in term of sex or that the population of Jericho in general comprised more males than females. Non-local ^87^Sr/^86^Sr ratios were found exclusively in two male individuals in this case. Irrespective of the intrinsic limitations of the strontium isotopic method, no isotopic evidence has been found indicating the mobility of any female individuals before their late adolescence in this study. There is no other obvious overall pattern according to sex in terms of burial type, or strontium, carbon and oxygen isotopic values.

## Discussion

The results of ^87^Sr/^86^Sr analysis on the measured environmental samples from Jericho, i.e., the local bioavailable strontium isotope interval of Jericho, agree with the predictions from our spatial mapping of ^87^Sr/^86^Sr ratios for the region (Fig. 4) for the geological province in which Jericho located, i.e., Legend E ranging from 0.707924 to 0.709260 (Dataset S2). Comparison of our human data to both of these datasets suggests that most individuals analyzed likely spent their childhood locally. According to the results of our ^87^Sr/^86^Sr analysis, only two biologically male individuals showed clear ‘non-local’ signatures falling beyond the baseline range. They are either from a different area within the Jordan Valley (Fig. 4, Legend E), perhaps they occupied a different part of the local landscape, or they came from further afield. The documented variability in bioavailable ^87^Sr/^86^Sr makes any discussion of provenance problematic (Fig. 4). When comparing the three human ^87^Sr/^86^Sr values which fall beyond the estimated baseline, the ratios of the M1 of JCH084 (0.708407) and of JCH061 (0.708497) fall in the range of Rendzina soils (consisted of Chalk/Marle) distributed in the Judean foothills, extending from 0.7084 to 0.7085, lower than the ranges reflecting the areas dominated by coastal alluvium (~ 0.7089), calcareous sandstone (0.7087 ± 0.0001) and Terra rossa soils (consisted of limestone and dolomite, 0.7086 ± 0.0003)^67^. Alternatively, when comparing them at the scale of datasets from individual sites, the three most closely matching locales are the sites of Wadi Yabis (0.708398–0.708582, 2SD), Hayonim Cave (0.708147–0.708483, 2SD) and Palestine Jerusalem (0.707879–0.708545, 2SD) (Dataset S4), though they are not contemporaneous to Jericho. JCH061’s M2 ^87^Sr/^86^Sr value of 0.709341 is the highest amongst all the data reported in our study, making it very possible that he came from the coastal region of the Eastern Mediterranean where marine sand and calcareous sandstone predominate (Fig. 4, Legend B, 0.708258–0.709966). To summarize the travel histories of the two non-local males recovered at PPN Jericho, they both may have been born somewhere outside Jericho possibly either within or beyond the Jordan Rift Valley. Later, individual JCH061 also lived in the coastal region based on the ratio of his M2. Nevertheless, we must be aware that there are still other possibilities for the variation in our data, including the potential of sourcing of food and water from different geologies by these individuals^75^. It is noteworthy that the M1 of individual JCH061 was heavily worn, seemingly due to certain crafting activities (SI Appendix Note S1). No special features of their burial contexts nor grave goods are distinctive from the other local inhabitants at PPN Jericho.

All other analyzed individuals generally fall within the local bioavailable strontium range for Jericho, despite some ratios being very close to the absolute limits of the defined local range (e.g., JCH042, 054, 075, 076, 097, Fig. 2). We take this to mean they were all raised locally (during the periods of life represented by the samples from the M1 and M2) within the Jericho area. However, the local expected ^87^Sr/^86^Sr range of Jericho spans the ranges of almost all the geological zones, not only the Southern Levant shown in Fig. 4, but also some areas in the Northern Levant such as the Amuq Valley^76^. As a result, movement between Jericho and areas with homogenous geological contexts cannot be excluded. Nevertheless, the extremely narrow distribution of ^87^Sr/^86^Sr ratios of the individuals from PPN Jericho, as shown in Fig. 2, may further support a high degree of homogeneity in the source region of their diet and water intake, which could be consistent with a local origin. The standard deviation among all the ^87^Sr/^86^Sr ratios reported, excluding the three outliers mentioned above, is 0.000058 (n = 49). Local human and faunal ratios typically vary by less than ± 0.0003^73^. Therefore, without evidence to the contrary, the data support the fact that no clear patterns of mobility were found in PPN Jericho, perhaps supporting the idea that sedentism had been achieved as early as the PPNA phase. This would be much earlier than at Nevali Çori, in Southeastern Anatolia, where the degree of mobility was still high in the early stages of the PPNB^15^. Nevertheless, this interpretation and comparison must remain cautious noting the limited sample size for each spatiotemporal group and requires higher resolution of the local strontium baseline and temporal changes in human ^87^Sr/^86^Sr, as well as the application of other proxies like lead isotope analysis for further insights.

Based on this assumption, all of the tested female individuals at PPN Jericho seem to have spent their childhood at the site. This is evidenced by JCH060, for example, with the ^87^Sr/^86^Sr ratios of her M1 (0.7080110), M2 (0.708018) and M3 (0.708020) all showing that she likely lived at Jericho her entire childhood, adolescence, and early adulthood, though travelling after these life stages cannot be excluded due to the inherent limitations of this method which targets early life. The epigenetic traits of human teeth from the PPNB site Kfar HaHoresh in the Southern Levant showed biological relationships between females and subadults, which is not the case between any males and subadults, indicating a possible matrilocal residence pattern^77^. On top of the potential mobility pattern reflected by the ^87^Sr/^86^Sr values in this study, with no female and only two male immigrants at PPN Jericho, the assumption that a matrilocal social organization may have existed at Jericho should not be excluded, although we must note that the evidence for male mobility is also limited in terms of sample size and this is only an assumption awaits further more solid evidence to verify. The two non-local male individuals identified on the basis of their ^87^Sr/^86^Sr values, JCH061 and JCH084. In some cases of multi-sampled individuals (i.e., JCH062, 060, 058, 042), the δ^13^C values of M1 are the highest which are supposed to result from weaning effect^78^, with exceptions of JCH061, 063 and 064. It is noteworthy that for individual JCH061, the M2 had a δ^13^C value 1.2‰ higher than its M1, which is a notably high intra-individual variability compared to other multi-sampled individuals measured that generally showed no detectable change or a variability of < 1‰ (i.e., JCH064, 063, 062, 060, 058, 042). This indicates that the diet of JCH061 during his late childhood, as represented by M2, might have incorporated more of a C_4_ or C_3_–C_4_ intermediate input (in the form of vegetation or animals consuming these vegetation), perhaps suggesting movement to a drier environment^79^. Additionally, the δ^18^O value of the M2 of JCH061 is lower than that of the M1, indicating that this individual likely moved somewhere further inland or at a higher altitude or latitude during the time reflected by the M2^80,81^, which also corresponds to its highest ^87^Sr/^86^Sr ratio as an outlier mentioned above perhaps indicating a wider mobility.

We grouped the plots of paired δ^13^C and δ^18^O isotopes of Jericho human teeth with their human/animal counterparts from the other sites dating prior to (Ain Mallaha dating to Natufian period) or approximately coevally with (Tell Qarassa North, Kharaysin, ‘Ain Ghazal, Beisamoun, el-Hemmeh and ‘Ain Jamman of PPN periods) Jericho (Fig. 3, Dataset S6)^37,82^. The δ^13^C values of Jericho are largely consistent with the corresponding values of human individuals from the other prehistoric sites in the Levant (i.e., ‘Ain Mallaha, Tell Qarassa North, Kharaysin, Beisamoun, ‘Ain Ghazal), reflecting a common dietary composition in terms of predominant consumption of C_3_ resources in the early Holocene of the Southern Levant. The Jericho values are, however, more dispersed which potentially suggests a likely diversified and broader diet for Jericho inhabitants in PPN times (Fig. 3). However, Jericho human exhibit higher δ^18^O values compared to those from ‘Ain Mallaha, Tell Qarassa North, Kharaysin, Beisamoun, ‘Ain Ghazal, sites whose ages range from the Natufian to PPNC periods. This is supported by a non-parametric Kruskal–Wallis test which shows significant differences between the δ^18^O values of Jericho and the other five groups (Dataset S6, p < 0.01; a Shapiro–Wilk test showed that the data was not normally distributed, W = 0. 95, p < 0.01). This difference could be linked to factors such as altitude, humidity and continental positioning that result in a wetter and warmer environment in Jericho compared to the other sites more inland or located at higher altitude (Figs. 1, 3). The δ^13^C and δ^18^O values of the plotted PPNB ungulates from el-Hemmeh and ‘Ain Jamman are much more scattered as the livestock pastures were proven to be influenced by seasonally directed husbandry strategies (e.g., vertical transhumance) resulting to very different patterning of their diet access from human^82^.

The results of sex determination on the randomly selected (meaning that the samples were not chosen with any special funerary context or obvious cultural/gender indication) individuals from PPN Jericho showed the human sex ratio at birth (SRB) is approximately equal to 1.59 (Male: Female = 27: 17), showing an imbalance. The natural ratio at birth between males and females in humans has been estimated to be within a narrow range of 1.07 to 1.03, slightly biased towards the male sex^83^. The sex ratio of a total ancient population can be affected by various factors^84^ such as war, sex-selective abortions, infanticide, aging, gendercide and so on. In archaeological cases, the factor of potential sex-specific burial practices is also worth considering. The Neolithic Demographic Transition model (NDT) argues that the advent of a sedentary lifestyle led to a rise in female fertility, along with an upsurge in both female and infant mortality rates^85^. If this was the case at Jericho, at this early phase of sedentism, they may have also experienced an attendant explosive mortality rate of women of childbearing age. The mortuary system in the Southern Levant during the PPN was complex with, for instance, many of the human remains either missing or represented only by a skull^86,87^. However, without more information on potential special burial areas for specific groups at PPN Jericho, the sex bias found in this study suggests the possibility of a specialized burial locale for females or other potentially particular groups. Last but not least, it must be pointed out that the sample size in this study is limited. Moreover, the local range of Jericho is similar to other regions in the larger area of the southern Levant. More samples with detailed contextual information and more future work on baseline variability is needed.

The lack of aDNA preservation does not allow us to determine if cross-cousin marriage, which was practiced at Basta^65^ and Ba’ja^15^, was also practiced at Jericho in the later PPNB. However, all existing evidence from Jericho seems to indicate a stable community, as reflected by the apparent lack of large-scale and/or structured mobility during youth, which lines up with the settlement stability mirrored by parallel sites in the Southern Levant where consanguineous endogamy within local communities has been demonstrated. It has been argued that an increasing permanence and occupation area of communities, including but not limited to co-residence, are evident in the Natufian and into the PPN (certainly in the PPNA), as indicated by the nature of monumental architecture at that time at sites such as WF16, Göbekli Tepe and Jerf el Ahmar^88–90^. Community efforts and rituals embodied in both mortuary practices and monumental buildings like the walls and tower of Jericho would also have further contributed to establishing a hold over an area with favorable resources^34^ and creating a local community with shared practices. Indeed, while a similar cultural framework can be identified across the whole Levant, distinctive characteristics of each regional locality and local community are emphasized in different areas. For example, skull rituals are commonly adopted in the PPN Levantine culture sphere, but they were executed differently in different sites/regions as reflected in the different use of plain skulls, plastered skulls and plaster statues at different sites^32^.

To summarize, the results of the multi-isotope analyses conducted here do not contradict the existence of a sedentary community at PPN Jericho with no evidence for large-scale migration into the community or structural mobility, although it should be noted that local values could also represent other areas of the Levant beyond Jericho, and therefore this interpretation should remain tentative. Many elusive issues of Jericho society remain unsolved: for example, the relationship of the Jericho community to other sites in the Southern Levant, the political organization of society at the inter-group and intra-community levels^91^, whether the concept of united community or/and households would have been even sharper for such a settlement with favorable living conditions and resources enclosed by the walls and how reproductive spheres were shaped and how they extended across space^92^. Considering the preservation issues for the collagen materials in the Levantine area, for example, we have failed in obtaining the δ^13^C and δ^15^N isotopic data from bone collagen and aDNA data which prevent us from further studies on the relevant diet and genomic history of populations during this time, researchers may have to rely more on approaches like elemental and isotopic analyses on excavated inorganic and bioapatite materials available and seek to undertake more tests on fresher samples from recent excavations. Nevertheless, this study provides a solid grounding for future multidisciplinary research that can shed further light on human societies across the PPNA and PPNB periods in the Southern Levant.

### Supplementary Information

Supplementary Information 1.

Supplementary Information 2.

## Data Availability

Sources for all downloaded data are presented in SI Appendix, Datasets S1–S6. The mass spectrometry proteomics data have been deposited into the ProteomeXchange Consortium (http://proteomecentral.proteomexchange.org) via the iProX partner repository^94^ with the dataset identifier PXD037215.
