# Supplementary material for: Isotopic and proteomic evidence for communal stability at Pre-Pottery Neolithic Jericho in the Southern Levant
Source: Sci Rep. 2023 Sep 29;13:16360. doi: 10.1038/s41598-023-43549-1 (PMC10542335; doi:10.1038/s41598-023-43549-1)
Supplement: Supplementary file 1 — Supplementary Information 1. [file 41598_2023_43549_MOESM1_ESM.docx]

**Isotopic and proteomic evidence for** **communal stability at Pre-Pottery Neolithic Jericho in the Southern Levant**

Xiaoran Wang, Baoshuai Zhang, Yufeng Sun, Tara Ingman, Stefanie Eisenmann, Mary Lucas, Erin Scott, Jana Ilgner, Gao Wu, Petrus le Roux, Xiaotong Wu, Xingxiang Zhang, Anchuan Fan, Patrick Roberts, Philipp W. Stockhammer

**Supplementary Material**

**Table of contents**

**Supplementary Notes**

**Note S1. Materials**

**Note S2.** **Methods**

**Note S2.1. Strontium isotope analysis**

**Note S2.2. Stable carbon and oxygen isotope analyses**

**S2.2.1. Methods of** **δ^18^O and δ^13^C isotope analyses**

**S2.2.2. Calibration and analytical uncertainty**

**Note S2.3. Proteomic Analysis**

**S2.3.1. Protein extraction**

**S2.3.2. nanoLC-MS/MS analysis**

**S2.3.3. Database search**

**S2.3.4. Deamidation analysis**

**Note S2.4. Stable carbon and nitrogen Isotope pretreatment**

**Supplementary Figures**

**Fig. S1. The osteological morphology of the M1 of individual JCH061.**

**Fig. S2. The environmental contexts where the modern plant samples were collected.**

**Table S6. Results of Kruskal-Wallis test comparison between the δ^18^O value of Jericho human teeth and the other parallel sites in the southern Levant.**

**Supplementary Tables (Datasets in** [**XLSX)**](https://www.pnas.org/highwire/filestream/1003550/field_highwire_adjunct_files/1/pnas.2022210118.sd01.xlsx)

**Table S1. Context and isotopic data of the samples studied**

**Table S2. The bioavailable local ^87^Sr/^86^Sr ranges in Fig. 4 (for the metadata and more details of the calculation, see Table S4)**

**Table S3. Failed measurements tested on bone samples recovered from Jericho for δ^13^C and δ^15^N values**

**Table S4. The published metadata and context information of the sites in the Levant used for constructing the bioavailable local ^87^Sr/^86^Sr ranges in Table S2 and Fig. 4**

**Table S5. The published metadata and context information of the sites in the Levant which are not included in Fig. 4 (beyond the scope of the map)**

**S1 Materials**

The detailed contextual information of the samples is listed in the Appendix Table S1. All samples were recovered from the site of Jericho, where the first excavations at the site were conducted by Charles Warren in 1868 ^1^. From 1952 to 1958, extensive excavations and research were led by Kathleen M. Kenyon ^2-4^. Subsequently, the Italian-Palestinian archaeological expedition carried out 13 seasons of excavation in 20 years (1997–2017) ^5,6^.

All skeletal material included in this study was excavated during the fieldwork under the direction of Kathleen Kenyon. During Kenyon’s fieldwork, Jericho was part of the territory of Jordan and all fieldwork followed the rules and permissions of the Jordanian state. As part of her team, she included the young German physical anthropologist Gottfried Kurth (*1912–†1990), who was responsible for the excavation and documentation of all human bone material found during the fieldwork. He worked closely together with Kenyon as well as the workmen, who gave him the nickname “Uncle Bone”. Including a physical anthropologist in the field in order to guarantee the best possible preservation and study of human bone material was novel and ground-breaking at that time. All bone material found during the excavation was kept - irrespective of the size of the fragment - and Kurth was to study this material as his contribution to the Jericho excavations. This treatment of the human bone material was rather unusual for that time, as many excavations of that time and later in many parts of the world usually discarded most of the bone material and only kept complete human skulls.

At the end of her work at Jericho, Kathleen Kenyon decided to hand over the human bone material to Kurth for further study. Consequently, the material was exported legally according to the rule of find division and brought to Germany where it should form the basis of Kurth’s anthropological collection at the University of Braunschweig, where Kurth had just accepted an academic position (“Hochschuldozent”) in 1965. During his life, Kurth tried to acquire funding for the study of the Jericho bone material, but he was unsuccessful, as reviewers considered the material too fragmented to be relevant for further study. When Kurth retired in 1977, the University of Braunschweig decided not to replace his position and wanted to discard Kurth’s bone collection. In order to preserve his collection, he handed it over to one of his last Ph.D. students, Olaf Röhrer-Ertl (*1940–†2019), whom he told to continue the analyses of the Jericho material. Shortly afterwards, Röhrer-Ertl received a position at the Bavarian Anthropological State Collection in Munich and, consequently, took the Jericho bones with him. The bone material was then stored in the rooms of his private apartment until his death in 2019. Then, the heirs’ community decided that the bones should be moved out of the apartment and rented a cellar for their storage. In the same year, Stockhammer was contacted by the heirs’ community via a lawyer. He was asked to help in the search for a future and sustainable storage place for human bones and to continue their scientific evaluation following the bequest and wishes of Kurth and the late Olaf Röhrer-Ertl. The permit for sampling was, therefore, issued by Eleonora Röhrer-Ertl, one of the daughters of Olaf Röhrer-Ertl as representative of the heirs’ community. Subsequently, an agreement was made with the anthropological collection of the University of Göttingen, where the bones were then brought for future storage after they had been sampled for bioarchaeological analyses in Munich. Since 2020, the bones have been kept under the custody of the University of Göttingen.

All information on the history and itinerary of the human bone material is based on the written estate of Röhrer-Ertl (private letters, notes, diaries etc.), which was scanned by the heirs’ and all scans were kindly forwarded to us. However, a comprehensive publication of the detailed contextual and anthropological analyses of Kurth and Röhrer-Ertl, which are preserved in this legacy, are beyond the scope of this publication.

Among the bone material selected by us for bioarchaeological study, it was interesting to note that among the teeth the M1 of individual JCH061 was heavily worn (Fig. S1), which is caused by different kinds of long-term habitual practice, such as subsistence or crafting activities.

In addition, to determine a baseline interval for the local ^87^Sr/^86^Sr signature at Jericho, we also sampled 1 archaeological animal tooth and 4 modern plant samples from different places in the vicinity of Jericho, with the coordinates all around 31.8 N, 35.4 E.


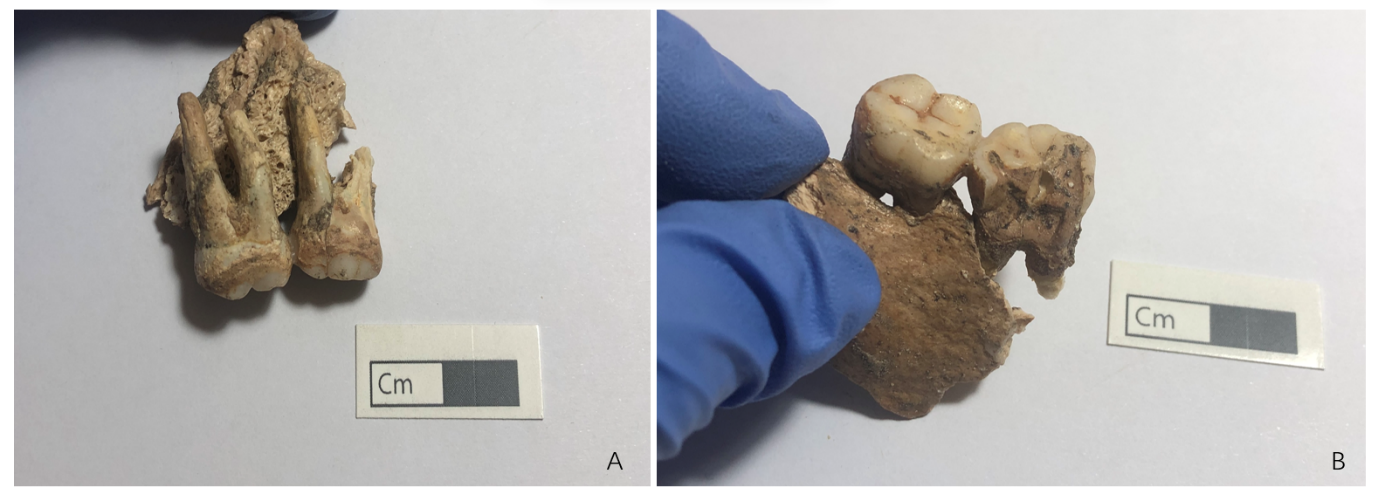


**Fig. S1.** The osteological morphology of the M1 of individual JCH061.

**
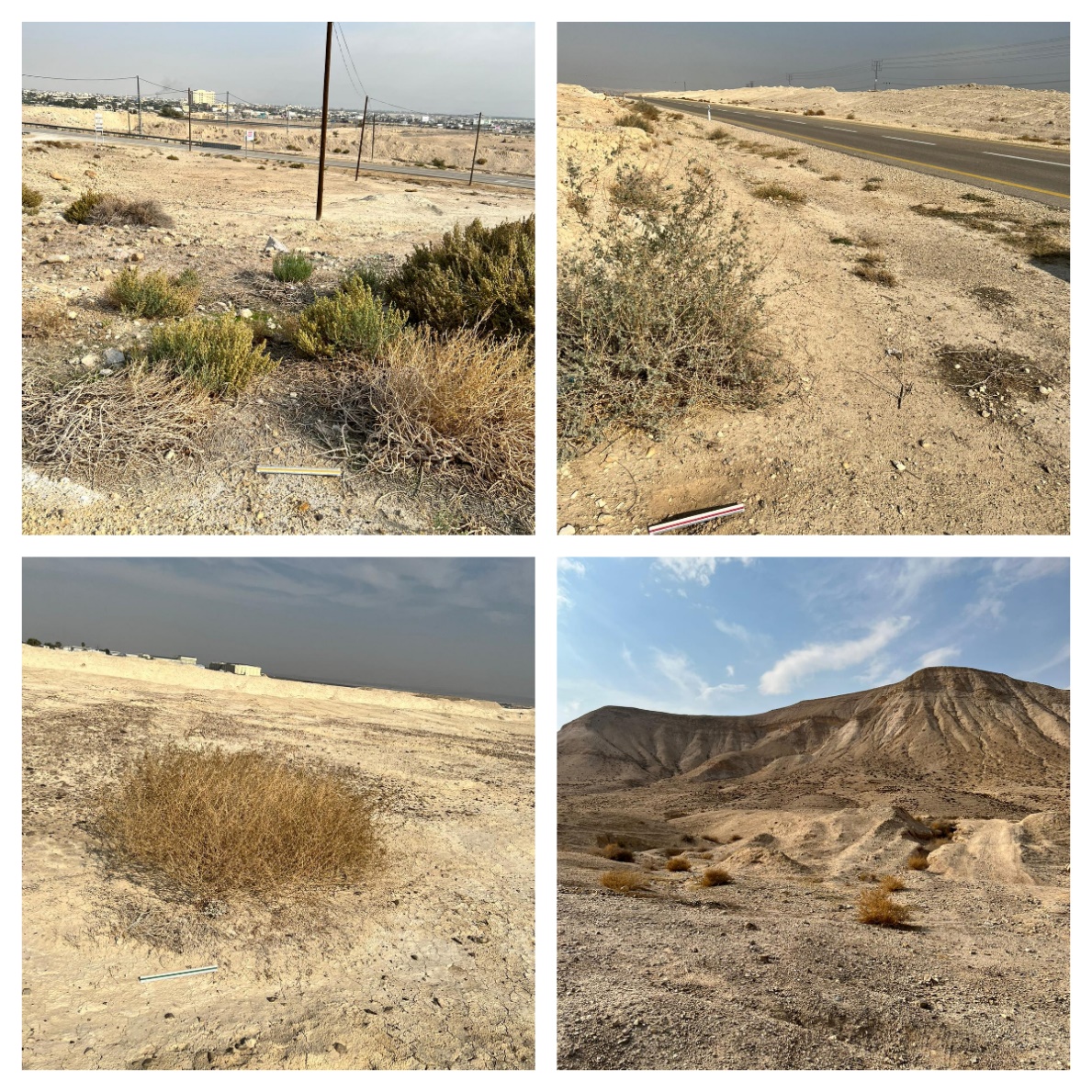
**

**Fig. S2.** The environmental contexts where the modern plant samples were collected.

**S2 Methods**

**S2.1. Strontium isotope analysis**

Analysis of ^87^Sr/^86^Sr of various bio- and geo-materials has been reported in many published studies from geographically and geologically varied regions across the Levant, both in comprehensive regional studies ^7,8^ and in local case studies where small batches of environmental materials from the target area serve as local base intervals (e.g., ^9-13^). These studies have enabled the construction of a reliable ^87^Sr/^86^Sr map for the southern Levantine area comparable with those have been built in other regions of the world, e.g., ^14-17^.

^87^Sr is a product of rubidium (^87^Rb) decay and varies in abundance within different types of bedrocks. The fact that rocks of different ages and Rb/Sr ratios have distinctive values provides the foundation for the use of strontium isotope ratios (^87^Sr/^86^Sr) to investigate human and animal mobility ^18,19^. The ratio of ^87^Sr/^86^Sr does not biologically fractionate as strontium moves through the food chain from the bedrock into the biosphere ^20-23^. ^87^Sr/^86^Sr in bone and teeth should thus reflect the bio-available strontium in the area where the human and animal lived during tissue formation ^21,24^. Tooth enamel is not remodeled after the formation of the crown, unlike bone which remodels throughout the life of individual with a turnover of strontium of ca. 26% per year ^25^. Thus, the ^87^Sr/^86^Sr ratio of dental enamel can serve as a good proxy for evaluating the locale where the individual was born and grew up ^21,26-29^. To establish local ranges (i.e., bioavailable local ^87^Sr/^86^Sr signature), different materials have been tested and evaluated such as bedrock, soil, underground water, spring water, and biological materials like plant, snails, modern and archaeological faunal teeth. This is due to the ways in which differential weathering of bedrock, different rooting heights, and other environmental factors can influence bio-available ^87^Sr/^86^Sr ^30-32^. It is essential to create a robust and reliable bioavailable base range for a regional iso-scape ^7,30,32-37^. Archaeological fauna, especially dental enamel from small mammals, provide an important material for constructing bioavailable local ^87^Sr/^86^Sr signature for an archaeological site ^26,35^.

All teeth in this study were sampled at the Stable Isotope Laboratory, Max Planck Institute of Geoanthropology (formerly the Max Planck Institute for the Science of Human History), Jena, Germany before being analyzed at the clean laboratory in the Department of Geological Sciences at the University of Cape Town. They were first cleaned using a sandblaster, and a groove was made along the buccal edge of the tooth in order to average the signal for the period of enamel formation ^26^. We obtained powdered samples of 20 mg from each specimen in Jena using a Dremel drill, and the samples were then shipped to Cape Town. Samples were dissolved in 2 mL 65% HNO_3_ in a closed Teflon beaker and placed for one hour on a hotplate at 140ºC. The samples were then dried down and re-dissolved in 1.5 mL 2M HNO_3_. Strontium separation chemistry followed methods discussed in ^38^. After separation, the solutions for each sample were dried, dissolved in 2 mL 0.2% HNO_3_ and diluted to 200 ppb Sr concentrations for strontium isotope analysis. Radiogenic ^87^Sr/^86^Sr ratios were measured using a Nu Instruments Nu Plasma HR MC-ICP-MS in the Department of Geological Sciences at the University of Cape Town. Sample values were corrected for instrumental mass fractionation using the exponential law and an ^86^Sr/^88^Sr ratio of 0.1194 ^39^, and isobaric ^87^Rb interference using the measured ^85^Rb signal and the natural Rb isotope ratio. All data presented here are referenced to bracketing analyses of NIST SRM987 (^87^Sr/^86^Sr reference value of 0.710255). Results for repeat analyses of an in-house carbonate reference material (NM95) processed and measured with the batches of unknown samples in this study gave an average ^87^Sr/^86^Sr ratio of 0.708911 (2s = 0.000012; n = 7) and are in agreement with long-term results for this in-house reference material having an average ^87^Sr/^86^Sr ratio of 0.708911 (2s = 0.000040; n = 414). Total procedural elemental Sr blanks agreed with typical values of < 250 pg in this facility, and therefore negligible.

The ^87^Sr/^86^Sr measurement of the plant samples was conducted at the Archaeometry Laboratory of the University of Science and Technology of China, Hefei. Approximately 10–25 mg of each lyophilized and chalked plant sample was weighed, and then 8M and 3M HNO_3_ were added for secondary purification, followed by the samples being dissolved at 200°C. Then, the solutions were centrifugalized, and the supernatant liquor was taken. In addition, the Teflon columns filled with Eichrom Sr-specific resin (mesh 100–150 μm) were rinsed with MilliQ water and 3M HNO_3_ multiple times to eliminate any Sr produced in the process of resin manufacturing. The purified Sr was extracted using cation exchange chromatography with a 3M HNO_3_ medium in the mobile phase. Then the Sr solutions were heated to dry and sealed after cooling. Part of the purified Sr was taken and diluted with 2% HNO_3_. The ^87^Sr/^86^Sr ratios were measured on a Neptune Plus MC-ICP-MS at the CAS Key Laboratory of Crust-Mantle Materials and Environments, School of Earth and Space Sciences, University of Science and Technology of China. The Sr carbonate standard NBS 987 yielded a value of ^87^Sr/^86^Sr = 0.710248 ± 0.000012 (2SD, n = 99).

**S2.2 Stable carbon and oxygen isotope analyses**

**S2.2.1 Methods of δ^18^O and** **δ^13^C isotope analyses**

The carbon in mammalian bioapatite is derived from blood bicarbonate and reflects carbon from all dietary components (protein, carbohydrates, fat) of the diet. Stable carbon isotope analysis of human tooth enamel carbonate, expressed as δ^13^C values relative to the V-PDB standard, has been applied extensively in palaeoecological and paleodietary studies ^40^. The patterning of carbon isotopic fractionation in plants is driven by the photosynthetic pathway (C_4_, C_3_ or CAM) through which the plant assimilate carbon ^41,42^, where C_3_-plants (Calvin cycle) discriminate more strongly against the heavier isotope of ^13^C than C_4_-plants (Hatch-Slack cycle) ^43^. Carbon isotope values can be impacted by water status, salinity, altitude, and more complicated environmental effects ^44-50^. On average, C_3_-plants have stable carbon isotope values of ca. –26‰ and C_4_-plants have values ~14‰ higher at ca. –12‰ ^42,51^. The δ^13^C of tooth bioapatite results from the whole diet and the consumption of various plant types during childhood and adolescence thus provides a good proxy for reconstructing the proportion of the C_3_/C_4_ plants utilized in diets of ancient inhabitant, e.g., ^52,53^, as well as animal dietary change associated with seasonal changes in pasture and human interference, e.g., ^54-56^. Absolute C_4_ consumers in pre-industrial contexts are demonstrated to have δ^13^C values that present ~14‰ higher than absolute C_3_ consumers (0 to +2‰ vs. ca. –12‰) ^57^. δ^13^C values that fall between C_3_ and C_4_ diet endmembers probably reflect mixed feeding.

Stable oxygen isotope (δ^18^O) analysis of skeletal remains has become a complementary tool for exploring human mobility and migration in bioarcheology. δ^18^O values are often utilized as a support for ^87^Sr/^86^Sr ratios when investigating human/animal mobility. These values indicate an individual’s water intake and differ according to local temperatures, altitude, seasonality, continentality and other environmental effects ^54,58^. The application of both ^87^Sr/^86^Sr and δ^18^O is often used to explore mobility patterns, including the observation of people buried in a different location to where they grew up. In order to explore these questions of mobility, we sampled both human remains from PPN Jericho and modern environmental contexts for ^87^Sr/^86^Sr analysis, allowing us to provide a new bioavailable ^87^Sr/^86^Sr range to fill a gap in existing ^87^Sr/^86^Sr baselines around this critical PPN site. δ^18^O values of mammal and human enamel bioapatite can be measured in carbonate (δ^18^O_ca_) or phosphate (δ^18^O_p_) which are derived from imbibed water and dietary water ^59,60^, which are in turn related to local environmental water ^61,62^. Given that dental enamel does not turnover after mineralization, δ^18^O in human dental enamel provides insight into the water source an individual relied on during tooth formation ^63^. δ^18^O measured in meteoric water varies across different geographic regions according to multiple variables such as distance from the ocean, precipitation, altitude, humidity, and latitude ^61,64-66^. δ^18^O values can therefore be applied to reconstruction of paleoclimate and paleoseasonality, human and animal lifetime provenance and cultural practices such as breastfeeding, animal husbandry and management practices, weaning behavior and culinary techniques, e.g., ^55,56,67^.

The sampled teeth were cleaned using a sandblaster to remove any external dirt. 7mg of enamel powder was taken and pre-treated using a protocol to remove any organic or secondary carbonate contaminates as followings. First we applied 1% sodium hypochlorite soaking the sample for 60 minutes, which was then rinsed by purified H_2_O three times and centrifuge, before 0.1 M acetic acid was added for 10 minutes, and the sampled was rinsed again in purified H_2_O for another three times ^68^. After reaction of the sample with 100% phosphoric acid, gases evolved from the samples were analyzed for stable carbon and oxygen isotopic composition using a Thermo Gas Bench II connected to a Thermo Delta V Advantage Mass Spectrometer at the Max Planck Institute of Geoanthropology (formerly the Max Planck Institute for the Science of Human History), Jena, Germany.

**S2.2.2 Stable isotope calibration and analytical** **uncertainty**

We compared the resulting values using a three-point calibration against International Standards (IAEA-603 (δ^13^C=2.46‰; δ^18^O=−2.37‰); IAEA-CO-8 (δ^13^C=−5.8‰; δ^18^O=−22.7‰); IAEA NBS 18 (δ^13^C =5.014‰, δ^18^O= -23.2‰). USGS44 (δ^13^C=−42.2‰) was run as an internal standard. The data from these standards suggest that the machine measurement error is c. ± 0.18‰ for δ^13^C and ± 0.38‰ for δ^18^O. An equid enamel standard was run to access systemic error (accuracy). Measurement bias due to systematic error was determined to be ±0.2 ‰ for *δ*^13^C and ±0.2 ‰ for *δ*^18^O.

**S2.3 Proteomic Analysis**

Because sex determination of the sampled individuals in the absence of both the complete skeletons for osteological identification and aDNA extraction for shotgun-genomic sex estimation is challenging, proteomics analysis was used, a technique that samples the amelogenin peptides on the enamel, which is the most accurate (and, indeed, the only) option to determine biological sex for the sampled individuals from PPN Jericho ^69,70^.

Recently, proteomic analysis of sex-specific amelogenin peptides in tooth enamel has been shown to provide reliable new approach to sex determination in archaeological studies ^69^. Amelogenin (AMEL) is the main protein for enamel formation, accounting for 90wt% of enamel matrix proteins (EMPs) in mature enamel ^71,72^. It is encoded by both chromosomes X and Y, with slight differences in amino acid sequences between the X and Y-derived AMEL, which allows sex estimation that enabled discussions of mobility along sex-based lines. Furthermore, dental enamel is the hardest, densest, and most calcified tissue in toothed vertebrates, which can protect proteins from external physical and chemical damage ^73^. A number of studies have shown the reliability of this method, especially in minors and poorly preserved individuals ^70,73-79^.

**S2.3.1 Protein extraction**

Most of the samples come from the residual enamel fragments of strontium isotope experiments, and a few samples directly etch the surface of the dental crown. The tooth surface was abraded using a dental burr to remove obvious surface contaminants. The enamel was washed with 3% H_2_O_2_ for 30 s and then rinsed with ultrapure water. 100 μL of 1.5M HCl was used to etch tooth enamel for 15 min, and the etching solution was collected in 1.5ml Eppendorf tubes. Desalting and elution were carried out according to Stewart et al ^69^. Finally, a 10 μL 60% acetonitrile/0.1% formic acid elution buffer with peptides was dried and centrifuged in a drying centrifuge. At the same time, a blank control was added to monitor possible contamination during the experiment.

**S2.3.2 nanoLC-MS/MS analysis**

The dried peptides were resuspended with 10 μl 0.1% formic acid solution and analysed by Easy-nLC1200 nanoflow liquid chromatography system (Thermo Fisher Scientific) coupled to a Q Exactive Plus mass spectrometer (Thermo Fisher Scientific). 3μL of the samples were loaded on a pre-column (Acclaim PepMapTM 100, 100μm×2cm, nanoViper 2PK, C18, 5μm, 100Å) at a flow rate of 300nL/min. After desalting, peptides were separated on an analytical column (Acclaim PepMapTM RSLC, 50μm×15cm, nanoViper, C18, 2μm, 100Å) with a linear gradient program: 3%−8% B in 3min，8%−28% B in 36min，28%−45% B in 10min，45%−90% B in 1min，90%−90% B in 10min (solvent A: 0.1% FA, solvent B: 80%CAN/0.1%FA).

The Q Exactive Plus mass spectrometer was operated in positive ion mode with a nanospray voltage of 1.8 kV and a source temperature of 275 ℃. Mass spectrometry data were acquired by automatic switching between MS1 scanning to up to 20 MS/MS scans (topN method). The target value of MS1 scanning was set as 1e6 in the m/z 350−2000 range with the maximum ion injection time of 50 ms and a resolution of 70000. Ion precursors were isolated with a window width of 1.6 m/z and MS/MS fixed first mass of m/z 110. Ion precursors were fragmented using the high-energy collision dissociation technique with the normalized collision energy of 27%. MS/MS scans were obtained with the resolution of 17500 and target ion value of 1e5 with the maximum ion injection time of 50ms.

**S2.3.3 Database search**

The AMELX gene encodes three amelogenin isoforms formed by alternative splicing: Q99217-1, Q99217-2 and Q99217-3 (Uniprot ID). The Q99217-1 isoform is considered the canonical amino acid sequence. The Q99217-2 isoform differs from the canonical sequence by deletion of the 19-34 fragment, and the isoform Q99217-3 by insertion of the ENSHAQAINVDRTAL sequence replacing Glu34 residue. As a result of alternative splicing, the AMELY gene produces two protein isoforms: Q99218-1 and Q99218-2 (Uniprot ID). The Q99218-2 isoform corresponds to the canonical amino acid sequence. The Q99218-1 isoform differs from the canonical sequence by deletion of the 19-34 fragment. Amelogenin isoforms Q99217-3 and Q99218-2 have the highest similarity. This study only focused on the sex of individuals: as such, two amelogenin isoforms Q99217-3 and Q99218-2, from UniProtKB were selected as retrieval databases, which could greatly improve the retrieval efficiency.

MS/MS data were analyzed with MaxQuant (version 1.6.0.1). Searches were conducted with unspecific digestion. Oxidation (M) and Deamidation (NQ) were set as variable modifications. The minimum peptide length was set to 6. Precursor mass tolerance was set to 10 ppm and fragment mass tolerance to 0.05 Da. The false-discovery rate of peptide spectrum matches was set to 0.01. Protein identifications were accepted if at least two unique peptide sequences were registered.

**S2.3.4 Deamidation analysis**

Python command line script provided by Mackie et al. 2018 ^80^ was used to calculate the deamidation percentage of glutamine (N) and asparagine (Q) in each sample to evaluate the reliability of ancient protein identification (https://github.com/dblyon/deamidation). The calculations were done separately for potentially original peptides and potential contaminants (human keratin). Briefly, the Maxquant "evidence. txt" file was used as the original file of the calculation. For each peptide containing N and Q residues, the ratio between the number of residues in the modified form and the total number of the residues was calculated, and this value was then multiplied for the intensity of the peptide. The values obtained were summed. The result was then divided by the total sum of all intensity values of the peptides in the modified and unmodified forms. 1000 bootstrap iterations were applied to calculate mean, standard deviation, and 95% lower and upper confidence intervals and to estimate the calculation error.

**2.4 Carbon and Nitrogen Stable Isotope Analysis of Bone (failed to get successful results）**

The human bone samples utilized in this study were prepared and analyzed at the Max Planck Institute for Geoanthropology (formerly the Max Planck Institute for the Science of Human History), Jena. Samples were taken from either long bones or ribs and prepared following the method described in Richards and Hedges ^81^. Samples were soaked in 0.5 M HCl to demineralize the collagen samples for two weeks with the HCl solution changed every 48 hours. Samples were rinsed three times with purified H_2_O then heated for 48 h at 70℃ in water acidified to pH 3, then removed from heat and immediately filtered through EZEE Filters. Finally, samples were freeze dried and weighed into tin capsules for analysis. Samples were analyzed in duplicate when sample size allowed, and results reflect their mean values. Mass spectrometry analysis for all new samples was conducted at the MPI-GEA Stable Isotope Laboratory using a Thermo Fisher Scientific FLASH 2000 HT Elemental Analyzer via a Thermo Fisher Scientific Conflo IV continuous flow interface coupled to a Thermo Fisher Scientific Delta V Advantage Isotope Ratio Mass Spectrometer. Collagen was successfully extracted in most cases, however, the results revealed that none of the samples fell within the required C/N ratio range or had a suitable standard deviation between runs (Table S3).

**Table S6. Results of Kruskal-Wallis test comparison between the δ^18^O value of Jericho human teeth and the other parallel sites in the southern Levant**

| **Level** | **- Level** | **Score Mean Difference** | **Std Err** | **Z** | **p-Value** | **Hodges-Lehmann** | **Lower CL** | **Upper CL** |
| --- | --- | --- | --- | --- | --- | --- | --- | --- |
| **Jericho** | **Ain Ghazal** | **33.0192** | **5.406954** | **6.10680** | **<.0001*** | **1.50000** | **1.20000** | **1.80000** |
| **Jericho** | **Beisamoun** | **27.8902** | **7.630860** | **3.65492** | **0.0003*** | **2.00000** | **1.50000** | **2.50000** |
| **Jericho** | **Ain Mallaha** | **27.3856** | **6.305786** | **4.34294** | **<.0001*** | **1.50000** | **1.00000** | **2.20000** |
| **Kharaysin** | **Beisamoun** | **4.3556** | **2.310141** | **1.88541** | **0.0594** | **0.50000** | **-0.10000** | **1.20000** |
| **Tell Qarassa North** | **Beisamoun** | **2.4545** | **3.917350** | **0.62658** | **0.5309** | **0.15000** | **-0.40000** | **1.10000** |
| **Kharaysin** | **Ain Ghazal** | **0.7828** | **3.575779** | **0.21893** | **0.8267** | **0.00000** | **-0.40000** | **0.50000** |
| **Kharaysin** | **Ain Mallaha** | **0.4444** | **2.512713** | **0.17688** | **0.8596** | **0.10000** | **-0.60000** | **0.80000** |
| **Ain Mallaha** | **Ain Ghazal** | **-0.3914** | **3.583433** | **-0.10923** | **0.9130** | **0.00000** | **-0.50000** | **0.50000** |
| **Tell Qarassa North** | **Ain Mallaha** | **-1.9571** | **3.591434** | **-0.54493** | **0.5858** | **-0.20000** | **-0.90000** | **0.60000** |
| **Tell Qarassa North** | **Kharaysin** | **-3.6010** | **3.592524** | **-1.00236** | **0.3162** | **-0.30000** | **-0.90000** | **0.40000** |
| **Beisamoun** | **Ain Mallaha** | **-3.8889** | **2.304956** | **-1.68719** | **0.0916** | **-0.60000** | **-1.30000** | **0.20000** |
| **Tell Qarassa North** | **Ain Ghazal** | **-5.2273** | **3.866154** | **-1.35206** | **0.1764** | **-0.30000** | **-0.70000** | **0.20000** |
| **Beisamoun** | **Ain Ghazal** | **-9.3273** | **3.906490** | **-2.38764** | **0.0170*** | **-0.50000** | **-1.00000** | **-0.10000** |
| **Kharaysin** | **Jericho** | **-28.4314** | **6.305874** | **-4.50871** | **<.0001*** | **-1.50000** | **-2.00000** | **-1.00000** |
| **Tell Qarassa North** | **Jericho** | **-29.9938** | **5.407581** | **-5.54661** | **<.0001*** | **-1.80000** | **-2.20000** | **-1.40000** |

**SI references**

1 Chevalier, N. Early excavations (pre-1914). *A Companion to the Archaeology of the Ancient Near East* **1**, 48-69 (2012).

2 Kenyon, K. M. Excavations at Jericho. *The Journal of the Royal Anthropological Institute of Great Britain and Ireland* **84**, 103-110 (1954).

3 Kenyon, K. M. Jericho and its setting in Near Eastern history. *Antiquity* **30**, 184-197 (1956).

4 Kenyon, K. M. Earliest Jericho. *Antiquity* **33**, 5-9 (1959).

5 Taha, H. Archaeological excavations in Jericho, 1995-2010. *Archaeological Heritage in the Jericho Oasis: A Systematic Catalogue of Archaeological Sites for the Sake of Their Protection and Cultural Valorisation*, 269-304 (2011).

6 Taha, H. Jericho: a Living History Ten Thousand Years of Civilization. (2010).

7 Hartman, G. & Richards, M. Mapping and defining sources of variability in bioavailable strontium isotope ratios in the Eastern Mediterranean. *Geochimica et Cosmochimica Acta* **126**, 250-264, doi:10.1016/j.gca.2013.11.015 (2014).

8 Moffat, I. *et al.* Bioavailable soil and rock strontium isotope data from Israel. *Earth System Science Data* **12**, 3641-3652, doi:10.5194/essd-12-3641-2020 (2020).

9 Perry, M. A., Coleman, D. & Delhopital, N. Mobility and exile at 2nd century AD Khirbet edh‐Dharih: Strontium isotope analysis of human migration in Western Jordan. *Geoarchaeology: An International Journal* **23**, 528-549 (2008).

10 Al-Shorman, A. & El-Khouri, L. Strontium isotope analysis of human tooth enamel from Barsinia: a late antiquity site in Northern Jordan. *Archaeological and Anthropological Sciences* **3**, 263-269, doi:10.1007/s12520-011-0065-0 (2011).

11 Arnold, E. R. *et al.* Isotopic Evidence for Early Trade in Animals between Old Kingdom Egypt and Canaan. *PLoS One* **11**, e0157650, doi:10.1371/journal.pone.0157650 (2016).

12 Gregoricka, L. A. & Sheridan, S. G. Continuity or conquest? A multi-isotope approach to investigating identity in the Early Iron Age of the Southern Levant. *Am J Phys Anthropol* **162**, 73-89, doi:10.1002/ajpa.23086 (2016).

13 Santana, J. *et al.* Multi-isotope evidence of population aggregation in the Natufian and scant migration during the early Neolithic of the Southern Levant. *Sci Rep* **11**, 11857, doi:10.1038/s41598-021-90795-2 (2021).

14 Wong, M. *et al.* A bioavailable baseline strontium isotope map of southwestern Turkey for mobility studies. *Journal of Archaeological Science: Reports* **37**, doi:10.1016/j.jasrep.2021.102922 (2021).

15 Scaffidi, B. K. & Knudson, K. J. An archaeological strontium isoscape for the prehistoric Andes: Understanding population mobility through a geostatistical meta-analysis of archaeological 87Sr/86Sr values from humans, animals, and artifacts. *Journal of Archaeological Science* **117**, doi:10.1016/j.jas.2020.105121 (2020).

16 Wang, X. & Tang, Z. The first large-scale bioavailable Sr isotope map of China and its implication for provenance studies. *Earth-Science Reviews*, doi:10.1016/j.earscirev.2020.103353 (2020).

17 Willmes, M. *et al.* Mapping of bioavailable strontium isotope ratios in France for archaeological provenance studies. *Applied Geochemistry* **90**, 75-86, doi:10.1016/j.apgeochem.2017.12.025 (2018).

18 Faure, G. & Powell, J. L. in *Strontium isotope geology* 1-8 (Springer, 1972).

19 Ericson, J. E. Strontium isotope characterization in the study of prehistoric human ecology. *Journal of human evolution* **14**, 503-514 (1985).

20 Graustein, W. C. & Armstrong, R. L. The use of strontium-87/strontium-86 ratios to measure atmospheric transport into forested watersheds. *Science* **219**, 289-292 (1983).

21 Montgomery, J. Passports from the past: Investigating human dispersals using strontium isotope analysis of tooth enamel. *Ann Hum Biol* **37**, 325-346, doi:10.3109/03014461003649297 (2010).

22 Rokita, E., Hermes, C., Nolting, H.-F. & Ryczek, J. Substitution of calcium by strontium within selected calcium phosphates. *Journal of crystal growth* **130**, 543-552 (1993).

23 Price, T. D., Swick, R. W. & Chase, E. P. Bone chemistry and prehistoric diet: strontium studies of laboratory rats. *American Journal of Physical Anthropology* **70**, 365-375 (1986).

24 Capo, R. C., Stewart, B. W. & Chadwick, O. A. Strontium isotopes as tracers of ecosystem processes: theory and methods. *Geoderma* **82**, 197-225 (1998).

25 Price, T. D., Manzanilla, L. & Middleton, W. D. Immigration and the Ancient City of Teotihuacan in Mexico: a Study Using Strontium Isotope Ratios in Human Bone and Teeth. *Journal of Archaeological Science* **27**, 903-913, doi:10.1006/jasc.1999.0504 (2000).

26 Alexander Bentley, R. Strontium Isotopes from the Earth to the Archaeological Skeleton: A Review. *Journal of Archaeological Method and Theory* **13**, 135-187, doi:10.1007/s10816-006-9009-x (2006).

27 Price, T. D. *et al.* Strontium isotopes and the study of human mobility in ancient Mesoamerica. *Latin American Antiquity*, 167-180 (2008).

28 Price, T. D. *et al.* in *Archaeology and bioarchaeology of population movement among the Prehispanic Maya* 119-132 (Springer, 2015).

29 Wright, L. E. Identifying immigrants to Tikal, Guatemala: defining local variability in strontium isotope ratios of human tooth enamel. *Journal of Archaeological Science* **32**, 555-566 (2005).

30 Maurer, A. F. *et al.* Bioavailable 87Sr/86Sr in different environmental samples--effects of anthropogenic contamination and implications for isoscapes in past migration studies. *Sci Total Environ* **433**, 216-229, doi:10.1016/j.scitotenv.2012.06.046 (2012).

31 Grimstead, D. N., Nugent, S. & Whipple, J. Why a standardization of strontium isotope baseline environmental data is needed and recommendations for methodology. *Advances in Archaeological Practice* **5**, 184-195 (2017).

32 Frank, A. B. *et al.* The geographic distribution of bioavailable strontium isotopes in Greece - A base for provenance studies in archaeology. *Sci Total Environ* **791**, 148156, doi:10.1016/j.scitotenv.2021.148156 (2021).

33 Bentley, R. A., Price, T. D. & Stephan, E. Determining the ‘local’ 87Sr/86Sr range for archaeological skeletons: a case study from Neolithic Europe. *Journal of Archaeological Science* **31**, 365-375, doi:10.1016/j.jas.2003.09.003 (2004).

34 Knudson, K. J. & Price, T. D. Utility of multiple chemical techniques in archaeological residential mobility studies: Case studies from Tiwanaku‐and Chiribaya‐affiliated sites in the Andes. *American Journal of Physical Anthropology: The Official Publication of the American Association of Physical Anthropologists* **132**, 25-39 (2007).

35 Price, T. D., Burton, J. H. & Bentley, R. A. The characterization of biologically available strontium isotope ratios for the study of prehistoric migration. *Archaeometry* **44**, 117-135 (2002).

36 Sillen, A., Hall, G., Richardson, S. & Armstrong, R. 87Sr/86Sr ratios in modern and fossil food-webs of the Sterkfontein Valley: implications for early hominid habitat preference. *Geochimica et Cosmochimica Acta* **62**, 2463-2473 (1998).

37 Budd, P., Montgomery, J., Barreiro, B. & Thomas, R. G. Differential diagenesis of strontium in archaeological human dental tissues. *Applied geochemistry* **15**, 687-694 (2000).

38 Pin, C., Briot, D., Bassin, C. & Poitrasson, F. Concomitant separation of strontium and samarium-neodymium for isotopic analysis in silicate samples, based on specific extraction chromatography. *Analytica Chimica Acta* **298**, 209-217 (1994).

39 Nier, A. O. The isotopic constitution of strontium, barium, bismuth, thallium and mercury. *Physical Review* **54**, 275 (1938).

40 Ambrose, S. H. & Norr, L. in *Prehistoric human bone* 1-37 (Springer, 1993).

41 Bender, M. M. Variations in the 13C/12C ratios of plants in relation to the pathway of photosynthetic carbon dioxide fixation. *Phytochemistry* **10**, 1239-1244 (1971).

42 Smith, B. N. & Epstein, S. Two categories of 13C/12C ratios for higher plants. *Plant physiology* **47**, 380-384 (1971).

43 Kohn, M. J. & Cerling, T. E. Stable isotope compositions of biological apatite. *Reviews in mineralogy and geochemistry* **48**, 455-488 (2002).

44 Sage, R. F. Why C4 photosynthesis. *C4 plant biology* **3** (1999).

45 Araus, J. L., Amaro, T., Zuhair, Y. & Nachit, M. M. Effect of leaf structure and water status on carbon isotope discrimination in field-grown durum wheat. *Plant, Cell and Environment* **20**, 1484-1494, doi:10.1046/j.1365-3040.1997.d01-43.x (1997).

46 Yousfi, S., Serret, M. D. & Araus, J. L. Shoot δ15N gives a better indication than ion concentration or Δ13C of genotypic differences in the response of durum wheat to salinity. *Functional Plant Biology* **36**, 144-155 (2009).

47 Araus, J. L., Cabrera-Bosquet, L., Serret, M. A. D., Bort, J. & Nieto-Taladriz, M. A. T. Comparative performance of delta(13)C, delta(18)O and delta(15)N for phenotyping durum wheat adaptation to a dryland environment. *Funct Plant Biol* **40**, 595-608, doi:10.1071/FP12254 (2013).

48 Yousfi, S., Serret, M. D. & Araus, J. L. Comparative response of delta13C, delta18O and delta15N in durum wheat exposed to salinity at the vegetative and reproductive stages. *Plant Cell Environ* **36**, 1214-1227, doi:10.1111/pce.12055 (2013).

49 Ellsworth, P. Z. & Cousins, A. B. Carbon isotopes and water use efficiency in C4 plants. *Curr Opin Plant Biol* **31**, 155-161, doi:10.1016/j.pbi.2016.04.006 (2016).

50 Flohr, P. *et al.* What can crop stable isotopes ever do for us? An experimental perspective on using cereal carbon stable isotope values for reconstructing water availability in semi-arid and arid environments. *Vegetation History and Archaeobotany* **28**, 497-512, doi:10.1007/s00334-018-0708-5 (2019).

51 Sage, R. F. The evolution of C4 photosynthesis. *New phytologist* **161**, 341-370 (2004).

52 Wright, L. E. & Schwarcz, H. P. Stable carbon and oxygen isotopes in human tooth enamel: identifying breastfeeding and weaning in prehistory. *American Journal of Physical Anthropology: The Official Publication of the American Association of Physical Anthropologists* **106**, 1-18 (1998).

53 Buzon, M. R. & Bowen, G. J. Oxygen and carbon isotope analysis of human tooth enamel from the New Kingdom site of Tombos in Nubia. *Archaeometry* **52**, 855-868 (2010).

54 Blumenthal, S. A. *et al.* Stable isotope time-series in mammalian teeth: In situ δ18O from the innermost enamel layer. *Geochimica et Cosmochimica Acta* **124**, 223-236, doi:10.1016/j.gca.2013.09.032 (2014).

55 Makarewicz, C. A. Sequential δ13C and δ18O analyses of early Holocene bovid tooth enamel: Resolving vertical transhumance in Neolithic domesticated sheep and goats. *Palaeogeography, Palaeoclimatology, Palaeoecology* **485**, 16-29, doi:10.1016/j.palaeo.2017.01.028 (2017).

56 Ventresca Miller, A. R., Haruda, A., Varfolomeev, V., Goryachev, A. & Makarewicz, C. A. Close management of sheep in ancient Central Asia: evidence for foddering, transhumance, and extended lambing seasons during the Bronze and Iron Ages. *STAR: Science & Technology of Archaeological Research* **6**, 41-60, doi:10.1080/20548923.2020.1759316 (2020).

57 Farquhar, G. D., Ehleringer, J. R. & Hubick, K. T. Carbon isotope discrimination and photosynthesis. *Annual review of plant physiology and plant molecular biology* **40**, 503-537 (1989).

58 Laffoon, J. E. *et al.* Investigating human geographic origins using dual-isotope (87Sr/86Sr, delta18O) assignment approaches. *PLoS One* **12**, e0172562, doi:10.1371/journal.pone.0172562 (2017).

59 Luz, B., Kolodny, Y. & Horowitz, M. Fractionation of oxygen isotopes between mammalian bone-phosphate and environmental drinking water. *Geochimica et Cosmochimica Acta* **48**, 1689-1693 (1984).

60 Luz, B. & Kolodny, Y. Oxygen isotope variation in bone phosphate. *Applied Geochemistry* **4**, 317-323 (1989).

61 Dansgaard, W. Stable isotopes in precipitation. *Tellus* **16**, 436-468 (1964).

62 Longinelli, A. Oxygen isotopes in mammal bone phosphate: a new tool for paleohydrological and paleoclimatological research? *Geochimica et Cosmochimica Acta* **48**, 385-390 (1984).

63 Pederzani, S. & Britton, K. Oxygen isotopes in bioarchaeology: Principles and applications, challenges and opportunities. *Earth-Science Reviews* **188**, 77-107, doi:10.1016/j.earscirev.2018.11.005 (2019).

64 Gat, J. R. & Dansgaard, W. Stable isotope survey of the fresh water occurrences in Israel and the northern Jordan Rift Valley. *Journal of Hydrology* **16**, 177-211 (1972).

65 Gat, J. R. Oxygen and hydrogen isotopes in the hydrologic cycle. *Annual Review of Earth and Planetary Sciences* **24**, 225-262 (1996).

66 Levinson, A. A., Luz, B. & Kolodny, Y. Variations in oxygen isotopic compositions of human teeth and urinary stones. *Applied Geochemistry* **2**, 367-371 (1987).

67 Roberts, P. *et al.* Fossil herbivore stable isotopes reveal middle Pleistocene hominin palaeoenvironment in 'Green Arabia'. *Nat Ecol Evol* **2**, 1871-1878, doi:10.1038/s41559-018-0698-9 (2018).

68 Ambrose, S. H. Preparation and characterization of bone and tooth collagen for isotopic analysis. *Journal of archaeological science* **17**, 431-451 (1990).

69 Stewart, N. A., Gerlach, R. F., Gowland, R. L., Gron, K. J. & Montgomery, J. Sex determination of human remains from peptides in tooth enamel. *Proc Natl Acad Sci U S A* **114**, 13649-13654, doi:10.1073/pnas.1714926115 (2017).

70 Buonasera, T. *et al.* A comparison of proteomic, genomic, and osteological methods of archaeological sex estimation. *Sci Rep* **10**, 11897, doi:10.1038/s41598-020-68550-w (2020).

71 Salido, E. C., Yen, P., Koprivnikar, K., Yu, L.-C. & Shapiro, L. The human enamel protein gene amelogenin is expressed from both the X and the Y chromosomes. *American journal of human genetics* **50**, 303 (1992).

72 Fincham, A. G. *et al.* Human developing enamel proteins exhibit a sex-linked dimorphism. *Calcified tissue international* **48**, 288-290 (1991).

73 Ziganshin, R., Berezina, N. Y., Alexandrov, P., Ryabinin, V. & Buzhilova, A. Optimization of method for human sex determination using Peptidome analysis of teeth enamel from teeth of different biological generation, archeological age, and degrees of Taphonomic preservation. *Biochemistry (Moscow)* **85**, 614-622 (2020).

74 Rebay-Salisbury, K. *et al.* Child murder in the Early Bronze Age: proteomic sex identification of a cold case from Schleinbach, Austria. *Archaeol Anthropol Sci* **12**, 265, doi:10.1007/s12520-020-01199-8 (2020).

75 Froment, C. *et al.* Analysis of 5000 year-old human teeth using optimized large-scale and targeted proteomics approaches for detection of sex-specific peptides. *Journal of proteomics* **211**, 103548 (2020).

76 Lugli, F. *et al.* Sex-related morbidity and mortality in non-adult individuals from the Early Medieval site of Valdaro (Italy): the contribution of dental enamel peptide analysis. *Journal of Archaeological Science: Reports* **34**, 102625 (2020).

77 Lugli, F. *et al.* Enamel peptides reveal the sex of the Late Antique ‘Lovers of Modena’. *Scientific reports* **9**, 1-8 (2019).

78 Wasinger, V. C. *et al.* Analysis of the preserved amino acid bias in peptide profiles of iron age teeth from a tropical environment enable sexing of individuals using amelogenin MRM. *Proteomics* **19**, 1800341 (2019).

79 Parker, G. J. *et al.* Sex estimation using sexually dimorphic amelogenin protein fragments in human enamel. *Journal of Archaeological Science* **101**, 169-180 (2019).

80 Mackie, M. *et al.* Palaeoproteomic profiling of conservation layers on a 14th century Italian wall painting. *Angewandte Chemie International Edition* **57**, 7369-7374 (2018).

81 Richards, M. P. & Hedges, R. E. Stable isotope evidence for similarities in the types of marine foods used by Late Mesolithic humans at sites along the Atlantic coast of Europe. *Journal of Archaeological Science* **26**, 717-722 (1999).
